# The prototype DynamiX camera system – a high-frame-rate high-dynamic-range hard X-ray integrating detector for fourth-generation synchrotrons

**DOI:** 10.1107/S1600577526006375

**Published:** 2026-07-16

**Authors:** Simon Knowles, Darren Ballard, Dominic Banks, Stephen Bell, Manuele Bettelli, Ivan Church, Alex Dainty, Adam Davis, Vishal Dhamgaye, Oliver Fox, Marcus French, Thomas Gardiner, Navid Ghorbanian, Josh Harris, Matt Hart, Aswathi Koorikkat, Matt Larkin, John Lipp, John Matheson, Tim Nicholls, Joseph Nobes, Mark Prydderch, Kawal Sawhney, Matthew C. Veale, Matthew D. Wilson, Silvia Zanettini

**Affiliations:** a UKRI Science and Technology Facilities Council (STFC) Didcot United Kingdom; b IMEM-CNR, Parma, Italy; c Diamond Light Source Didcot United Kingdom; d Department of Chemistry University of Warwick Coventry Warwickshire United Kingdom; e Due2Lab S.r.l, Scandiano (RE), Italy; Brazilian Synchrotron Light Laboratory, Brazil

**Keywords:** X ray detectors, high dynamic range, fourth-generation synchrotron instrumentation, CdZnTe, integrating detectors

## Abstract

The development of fourth-generation synchrotrons, including the Diamond-II upgrade, promises 10–100× flux increases, reaching up to 10^12^ photons s^−1^ mm^−2^ at the detector, across a broad range of energies from 20 to 100 keV. To exploit fully these impressive photon fluxes and high X-ray energies, readout chips must achieve high frame rates and dynamic ranges, while the use of high-*Z* sensor materials is essential. To address these challenges, the UK’s Science and Technology Facilities Council has developed DynamiX, a test structure for a novel two-stage charge cancellation circuit on a 65 nm CMOS process with a dynamic range from single photon(s) per pixel per frame to >9000 photons per pixel per frame (10^11^–10^12^ photons s^−1^ mm^−2^) at 20 keV photon energy. The application-specific integrated circuit has 16 ×16 pixels on 110 µm pitch and is hybridized with 2 mm thick Redlen high-flux cadmium zinc telluride (HF-CdZnTe). Data are read out at 534000 frames per second over a 14 Gbps serialiser and frames are assembled and saved with a custom data acquisition system. Measurements were made on the Diamond Light Source (DLS) B16 Test Beamline using monochromatic X-ray beams of different sizes and energies to evaluate the detector performance. A sub-pixel beam of size ∼60 µm × ∼15 µm was used to probe pixels to measure single photons with a noise performance of σ = 5.7 ± 0.1 keV. These single photons are used to calibrate the test pulse and pixel cancellation packet sizes. The linearity of the detector response under increasing flux was measured from <1 photon per pixel per frame to ∼10^9^ photons s^−1^ mm^−2^ at 20 keV with an r.m.s. linearity of 6.2%. A polychromatic X-ray set was used to reach higher fluxes of ∼3 × 10^10^ photons s^−1^ mm^−2^ (20 keV equivalent), yielding an r.m.s. linearity of 3.2%. Finally, the full sensor area was used to image a rotating slitted disc at 534000 frames per second.

## Abstract 2

This paper presents the first results from the DynamiX X-ray imaging detector developed by the UK’s Science and Technology Facilities Council for a new generation of photon light sources. Operating at a continuous frame rate of 534 kHz and with a per-pixel per-frame dynamic range of roughly five orders of magnitude, this work is the first step in delivering a transformative new imaging technology.

## 1. Introduction

Synchrotrons around the world are upgrading to brighter diffraction-limited storage rings (Diamond, 2019 ▸; Raimondi, 2016 ▸). The capabilities of these upgraded facilities represent a challenge for existing X-ray imaging detectors due to the need to operate at extremely high fluxes while also reliably recording data with high spatial and temporal dynamic range (for example in crystallography). Existing silicon-based sensors are known to experience radiation damage at high fluxes, for example (Zhang, 2013 ▸), and at X-ray energies above 15 keV are effectively transparent, limiting their application on fourth-generation synchrotron beamlines operating at higher energies. Higher-*Z* sensor materials, such as cadmium zinc telluride (CZT), GaAs or CdTe, have higher stopping power and are therefore essential for imaging at these X-ray energies.

Most hybrid pixel detectors in use at synchrotrons operate in photon-counting mode, for example EIGER2 (Donath *et al.*, 2023 ▸) and PILATUS4 (Donath *et al.*, 2025 ▸). However, they are limited by their maximum count rate at <2 × 10^9^ photons s^−1^ mm^−2^ due to pileup (Fröjdh *et al.*, 2024 ▸), where multiple photons arrive simultaneously but are counted only once. They also cannot operate in pulsed mode.

An alternative approach is integrating detectors, where the charge created by X-rays in the sensor material is stored on large feedback capacitors in each pixel. The size of these feedback capacitors dictates the dynamic range of each of the pixels of the detector, with some systems opting for fixed gain (Hart *et al.*, 2012 ▸) and others making use of gain switching (Mozzanica *et al.*, 2016 ▸; Allahgholi *et al.*, 2019 ▸). However, the science needs of the photon science community require a new generation of detectors with improved spatial resolution, operating at higher frame rates and with improved dynamic range.

One option for addressing this challenging specification is to use more advanced and smaller silicon processes that offer the potential for more complex electronics in each pixel. However, the size of the feedback capacitors required for a wide dynamic range does not scale with the size of the silicon process.

One approach for high-flux integrating detectors which avoids the need for large capacitances is current mirroring, used on FASPAX (Zimmerman & Junnarkar, 2019 ▸), but the required DC bias current introduces effects which are challenging to manage.

An alternative approach is a charge cancellation architecture, where charge packets of opposite polarity to the input signal are applied to the front end whenever the signal reaches a set threshold, and the number of cancellations is counted. This charge packet cancellation frequency is orders of magnitude faster than the frame rate. This inherently digitizes the signal immediately and has been successfully used by MM-Pad (Tate *et al.*, 2013 ▸; Philipp *et al.*, 2020 ▸; Gadkari *et al.*, 2022 ▸) from Cornell University and Area Detector Systems Corporation, and in the prototype XIDER application-specific integrated circuits (ASICs) from ESRF and Heidelberg University (Fajardo *et al.*, 2020 ▸; Collonge *et al.*, 2024 ▸). In this work, results from the DynamiX camera system are presented, which uses this charge cancellation architecture but in a two-stage variant to extend, in the lower part, the dynamic range.

## 2. DynamiX ASIC architecture

### 2.1. Overview

**Figure 1:**
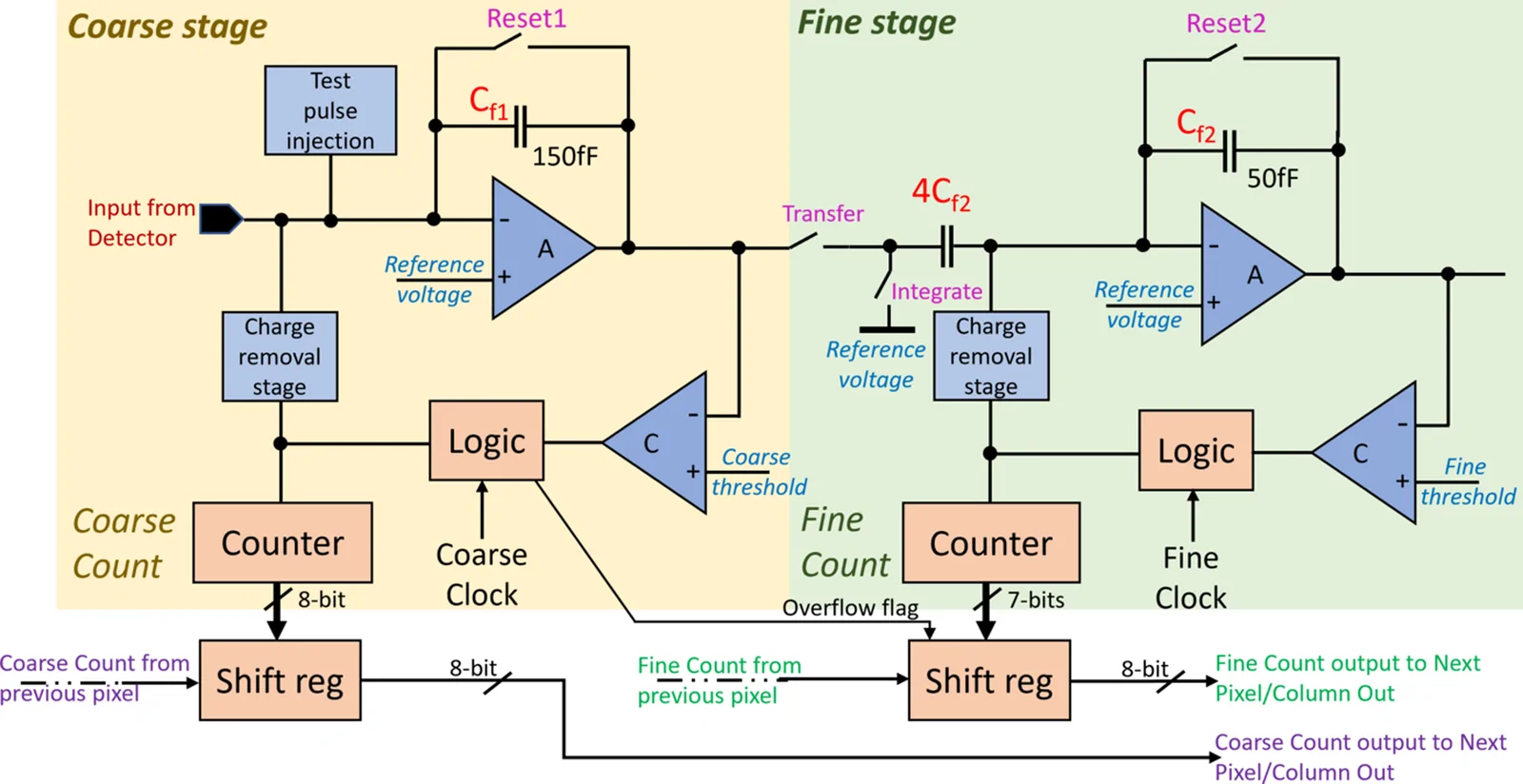
The two-stage charge cancellation architecture of the DynamiX ASIC pixel. The three reference voltages shown are equal.

Fig. 1 ▸ shows the architecture of a pixel in the DynamiX ASIC, which is an exclusively electron-collecting design. The two stages operate in a pipelined architecture, with the fine stage working after the coarse stage for a given frame. The detector is designed to work for 20–100 keV photons (25 keV typically).

**Figure 2:**
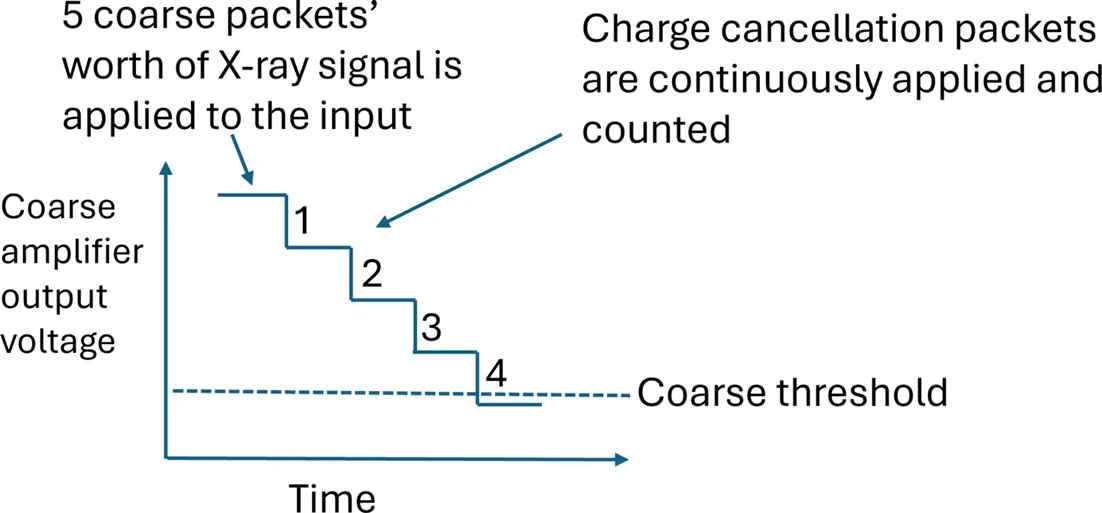
An illustration of charge packets being applied to the front end and cancelling the input signal below the coarse-stage threshold. In this case, a coarse count of ‘4’ is returned (the remaining charge is passed to the fine stage and counted there).

Charge on the pixel is converted to a voltage on the ‘coarse-stage’ pre-amplifier feedback capacitance. A comparator monitors the output of this amplifier. When it is above a customisable threshold, a ‘charge cancellation packet’ (of opposite sign to the incoming charge and of customisable size) is injected at the front-end input node, incrementing an 8-bit counter and reducing the voltage on the amplifier output. Cancellation packets are applied at 166 MHz, with a typical size of 750 keV equivalent (customisable between ∼0.5 keV and ∼2000 keV), and counted continuously during the frame if, and as long as, the output is above threshold. This process is shown in Fig. 2 ▸.

The residual charge in the coarse stage at the end of the frame results in a voltage at its output. The voltage difference compared with the reference voltage is then amplified by a factor of four by a transfer capacitor and the fine-stage feedback capacitance. A similar cancellation process then happens at 83 MHz with smaller charge packets of typical size 6 keV (customisable between <0.5 keV and ∼25 keV) and a 7-bit counter is incremented. In parallel to the fine-stage cancellation, the coarse stage begins integration and cancellation of the next frame of data. The coarse and fine counter values are therefore out of alignment by one frame, but this is corrected in the data acquisition (DAQ) system before the data are written to disk.

The coarse and fine thresholds and packet sizes are set globally by 12-bit digital-to-analogue converters (DACs), but there is significant offset between pixels in the reference voltage of the fine-stage amplifier, mostly due to mismatch in the reference buffers in the pixel. There is no trimming in the pixels to manage mismatch. This means the amplifier baselines are significantly different, which means dynamic range in the fine stage is lost in some pixels to bring all pixels into range. The threshold dispersion between the comparators, the comparators themselves and the pixel buffers also contribute to this reduced dynamic range. This will be corrected in the next version of the ASIC, with larger transistors to reduce the effects of mismatch, and per-pixel trim of the baselines and thresholds.

If the output of the coarse stage is *not* below the coarse threshold at the end of the cancellation window, then this means that it was not possible to remove all the charge delivered to the pixel in the frame time, because a very large signal was delivered to the pixel and/or signal arrived late in the frame. In this case, the ‘overflow’ flag is set, signifying that the returned coarse count value is not reliable, and these frames can be discarded in firmware or software.

For the chosen ideal size of coarse and fine packets, the total equivalent fine counts *T* is given by
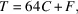
where *C* and *F* are, respectively, the returned coarse and fine counter values for that frame.

The coarse, overflow and fine counter values (16 bits total per pixel) of each pixel are output in turn and collated into two serialisers on the periphery of the ASIC. Each serialiser consumes the data from a 16 row × 8 column section of the ASIC. The pixel data are converted to 64B/66B Aurora encoded data and output at 14 Gbps. The serialiser has inbuilt test modes to output pseudo-random bit sequence (PRBS) data.

### 2.2. Detailed timing description

The signals that control the front end are set using a programmable waveform sequencer in the ASIC, allowing the timings to be fine tuned. Using the Diamond Light Source (DLS) storage ring RF clock (499.68 MHz) as the master clock, one frame is 1873 ns (936 clock cycles). The timing resolution of the sequencer is 1 cycle (∼2 ns).

In the following, the timing sequences of the coarse and fine stages are described consecutively, although it should be remembered they occur simultaneously in a ‘pipelined’ fashion. The frame begins with a reset of the coarse-stage amplifier (by closing Reset1) and a reset of the counter values. The coarse cancellation clock is then enabled, and charge is integrated and cancelled in the coarse stage. The effective integration window is around 1830 ns (depending on the length of the active reset applied). Towards the end of the frame, the coarse cancellation clock is disabled and the Transfer switch is then closed, so that the transfer capacitor (4C_f2_) tracks the voltage of the coarse-stage amplifier output. During this time, the coarse count value is loaded into the shift register and the comparator status of the coarse stage is captured. The Transfer switch is then opened at the end of the frame, and the transfer capacitor holds the final voltage output of the coarse-stage amplifier. Simulations suggest a minimum of two clock cycles are needed to allow the coarse-stage amplifier output to settle after a cancellation before the Transfer swich is opened.

At the start of the next (pipelined) frame, the fine stage comes out of reset (Reset2 is opened) and the Integrate switch is simultaneously closed, integrating the transferred charge onto the fine-stage feedback capacitance with a voltage gain of 4. Around 70 ns are allowed for this transfer. The fine-stage window for charge cancellation is then enabled until nearly the end of the frame, but runs only for the time required to convert the amplified residue to a 7-bit value. The fine stage is then reset, during which time the fine-count value is transferred to the shift register.

The global readout of 16-bit data from each shift register in the pixel is commenced after the load command. The data are read out per pixel column in parallel and concurrently when the pixels are in operation, so there is no dead time in the readout.

## 3. DynamiX detector system overview

**Figure 3:**
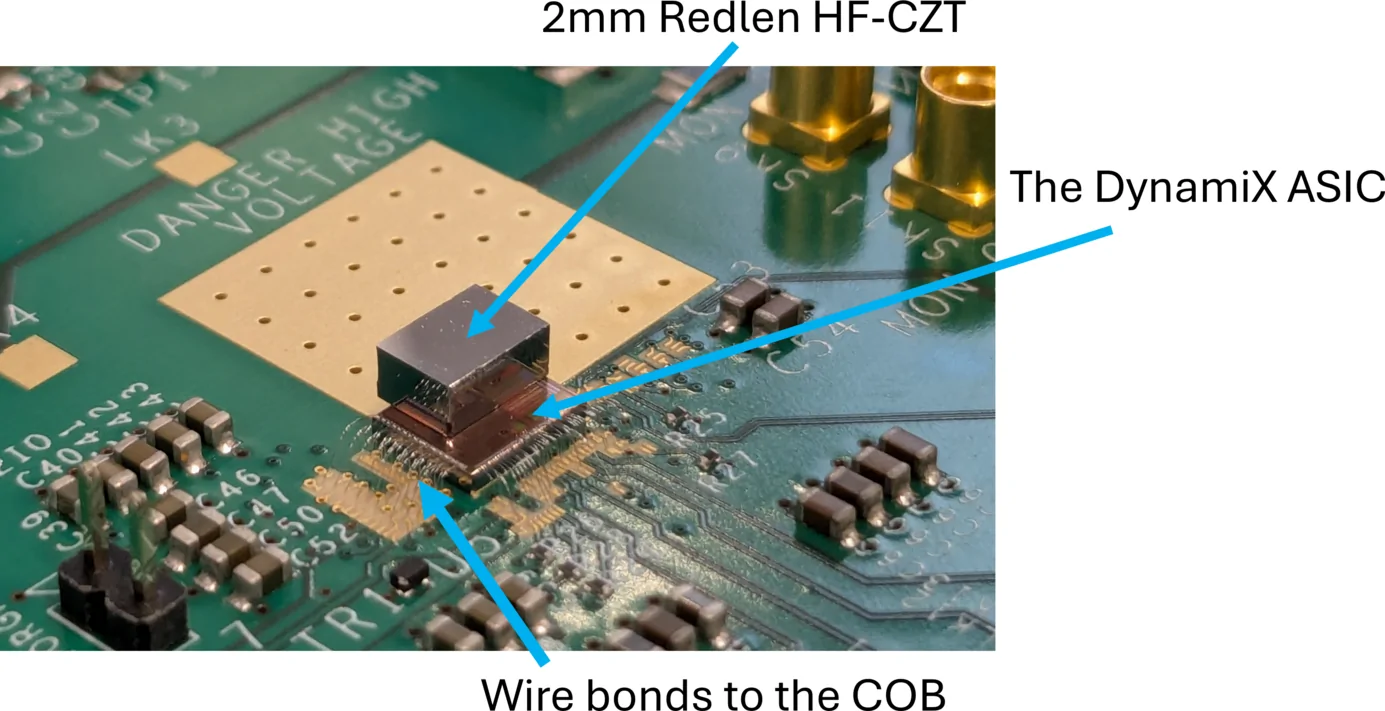
The DynamiX ASIC hybridized to high-flux CdZnTe and wire-bonded to the chip on board.

The DynamiX ASIC is gold-studded and silver-epoxy flip-chip bonded to Redlen high-flux CdZnTe (HF-CdZnTe, CZT). The CZT anodes were first reprocessed by Due2Lab following the procedure developed in collaboration with IMEM-CNR (Bettelli *et al.*, 2023 ▸) and then in-house bonded to the ASIC in the STFC Rutherford Appleton Laboratory (RAL) Cleanroom (Schneider *et al.*, 2015 ▸). The ASIC is then wire-bonded to a custom STFC COB (chip on board). The ASIC, which has been hybridized to CZT and wirebonded to the COB, is shown in Fig. 3 ▸.

**Figure 4:**
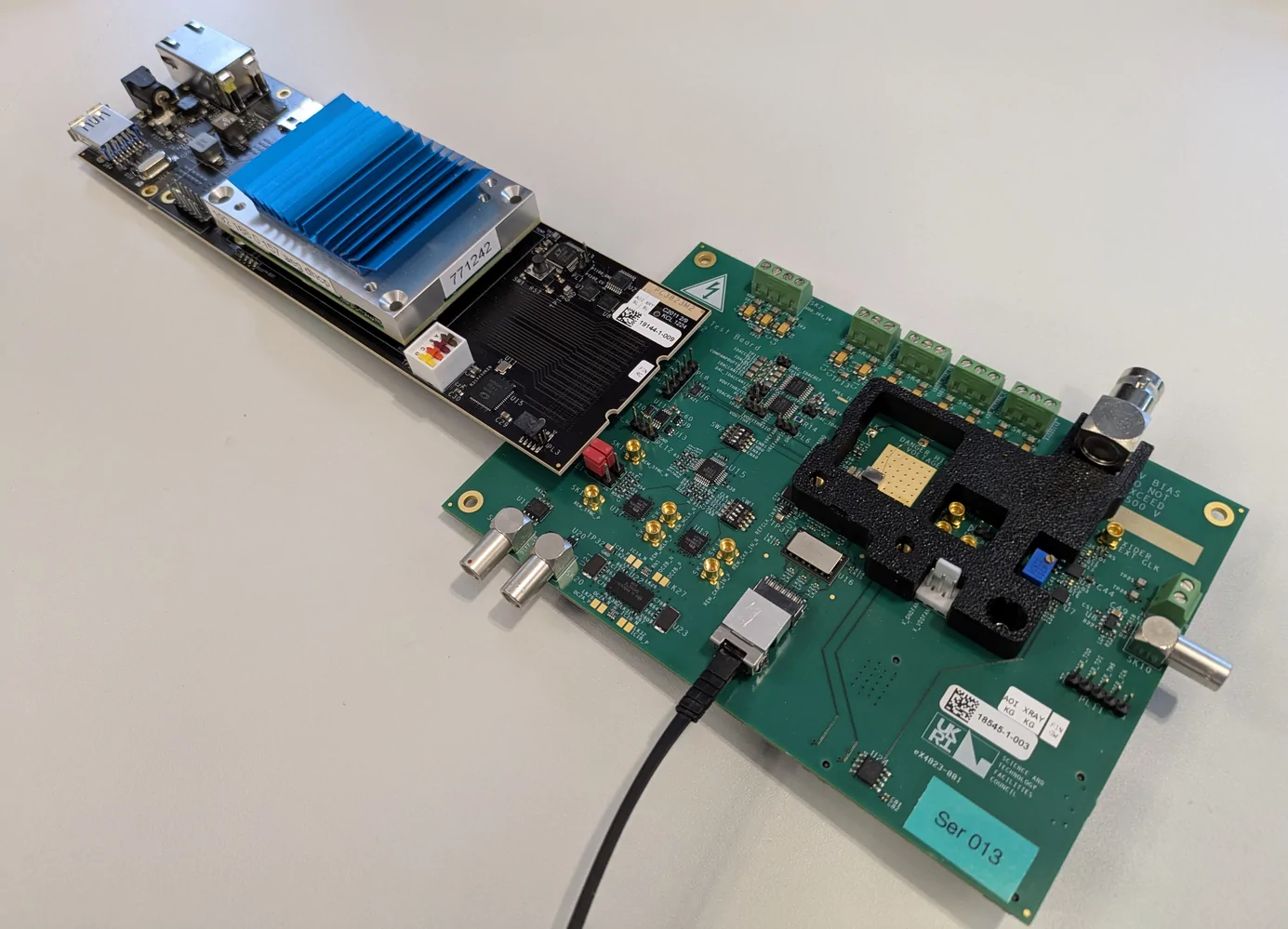
The green DynamiX COB (right-hand PCB) connected to the black LOKI control board (left-hand PCB). The ASIC itself is surrounded by the black protective cover in the centre of the COB.

The DynamiX COB supplies the ASIC digital and analogue power; allows monitoring of ASIC voltages/currents with external ADCs; provides an interface to external clocks and triggers (*e.g.* from a beamline); provides a connection to an externally-supplied HV bias; and interfaces the high-speed 14 Gbps serialiser with a fibre optic cable to the DAQ system. The COB connects to the LOKI control PCB, a custom board with a Xilinx ZynqMP SoM (system on module), designed with the versatility to control multiple detector systems with different requirements, including the HEXITEC-MHz ASIC (Veale *et al.*, 2023 ▸). The LOKI board operates the ASIC control interface and generates clocks for the ASIC (unless they are taken from the beamline and routed directly into the COB), serves a web front-end UI interfacing with Python control software, and allows interfacing with the I2C devices on the board, including the Firefly through which the high-speed serialized data are output over fibre optic cable. The COB and LOKI board are shown in Fig. 4 ▸.

Serialized data from the ASIC are output over two ribbon bonds at 14 Gbps using Aurora 64B/66B encoding and routed to a retimer IC. The retimer allows inspection of the eye diagram quality of the serialized data and a measurement of the bit error rate of the PRBS mode on the serialiser. From there, the data are sent over fibre optic link to a 200G transceiver on an Alpha Data card, where the data are decoded, assembled into frames, converted into UDP packets and then output from a 100G transmitter. The frames are finally received on a 100G network interface card (NIC) and saved as h5 files onto NVME drives using a custom software framework called *Odin-Data* (Yendell *et al.*, 2018 ▸), which utilizes kernel bypass techniques in *DPDK* (Data Plane Development Kit). The custom GUI, using LOKI and *Odin-Data*, allows users to watch a live view of what the detector is seeing, updated every second, or to request captures of any numbers of frames. The firmware and software robustly allow saving datasets of more than 100 million frames at 534000 frames per second.

## 4. Methods

### 4.1. Calibration methodology of DynamiX

The calibration methodology used in this work first calibrates the fine count size into kiloelectronvolts, followed by a fine counts to coarse counts cross-calibration. This is achieved by carefully adjusting the thresholds and charge cancellation packet sizes for both the coarse and fine stages. These four parameters are global to all pixels.

The fine stage is calibrated first, so the coarse stage is effectively ‘disabled’ by setting the coarse threshold to the maximum and delivering moderate input signals. Therefore, the coarse threshold is not exceeded and hence no charge cancellation packets are applied, so all the input charge from the pixel is passed to the fine stage as residue.

The per-pixel spectrum produced by histograms of multiple frames is used to calibrate the fine-stage charge cancellation packet size. The spacing (in fine counts) between one- and two-photon peaks of a fixed energy gives the calibration for fine counts to kiloelectronvolts. By changing the fine-stage charge cancellation packet size (*i.e.* the register DAC code), the desired fine counts to kiloelectronvolts ratio can be set. Note that this spectrum will have some offset from 0 fine counts = 0 keV before the fine threshold is correctly set, with some dependence on leakage current and exposure time.

To reduce this offset, the fine threshold is then swept in the dark (*i.e.* with no X-rays applied) to find the threshold value which corresponds to 0 fine counts ≃ 0 keV. As a simple approximation, we use the threshold value at which half of all frames have 0 fine counts, which corresponds to setting the threshold at the amplifier reference voltage. The other half of the frames will give a count ≥1 fine count, so we may overcount on some frames. The amount of overcounting is determined by the fine packet size and the noise on the reference voltage and threshold. This possible overcounting will be addressed in future work, for example by slightly raising the fine threshold, once the linearity of the test pulse for low currents has been further characterized, as would be needed to inject very small amounts of charge to calibrate the fine threshold.

Once the fine threshold and packet size have been calibrated, the coarse stage is calibrated using the test pulse in the ASIC. The charge delivered by the test pulse may be set either by varying the amplitude of the pulse (the current) or the duration for which the pulse is on.

It is essential that only ‘positive’ residue is passed to the fine stage and that the coarse stage is not ‘over-cancelled’ below the baseline, since this ‘negative’ residue cannot be corrected for in the fine stage. However, by setting the coarse threshold higher than the size of the coarse cancellation charge packet, there is always some residue passed to the fine stage which *can* be correctly counted. A target calibration of 1 coarse count = 64 fine counts is chosen, as this allows overlap between the coarse and fine stages (*i.e.* the maximum 7-bit range of the fine stage is two coarse counts).

A parameter sweep is used to find an amplitude–duration combination for a test pulse equivalent to 64 fine counts (*i.e.* 1 target coarse count) to calibrate the coarse stage. While injecting this test charge, the coarse threshold is swept. When the comparator threshold is too low, the charge cancellation pump will be triggered every frame. When it is too high, the charge pump will never be triggered. The threshold is then set a little higher than that which gives a 50% probability of triggering, to prevent these over-cancellations which would pass negative residue to the fine stage

The coarse packet size is then calibrated. The charge-cancellation packet size is varied so that each cancellation removes the correct amount of charge, equal to 64 fine counts. When the packet size is too small, more charge packets are required to remove the same total amount of charge. When the packet size is larger, fewer charge packets are required. Charge equivalent to *n* coarse packets is injected. The goal is then, by sweeping the coarse-stage charge packet size, to find the largest charge packet size which yields a count value of *n* − 1. This gives only a good approximation of the correct charge packet size; the calibration is then verified and optimized by injecting linearly increasing amounts of charge per frame, and verifying that the total equivalent fine counts increase linearly with the correct gradient.

Due to mismatch, it is only possible to have one pixel optimally calibrated with this procedure. However, with the introduction of per-pixel trims, a similar calibration procedure would be performed, with the per-pixel trim for each pixel set at the relevant step in the above procedure. It would be necessary to do this iteratively – first to find the appropriate global DAC values (the two thresholds and charge cancellation packet sizes) such that all pixels fall within the trim range, then setting the trim for each pixel in parallel. In this way, the dynamic range of each pixel can be maximized.

Alternative calibration strategies for DynamiX are also possible, for example by setting the thresholds and packet sizes to reasonable but non-optimized values, then using X-rays to determine the offsets and gains. While this approach is viable, it relies more heavily on post-processing and does not inherently guarantee optimal use of the dynamic range. In particular, certain constraints must always be satisfied: the coarse threshold must exceed the coarse packet size to avoid over-cancellation (which cannot be corrected offline), and the fine-stage range must at least match the coarse threshold to prevent saturation of the fine counter (which also cannot be corrected offline). Additionally, selecting a coarse threshold significantly larger than the packet size leads to under-utilization of the coarse stage and reduced overall dynamic range.

### 4.2. Experimental setups

The 2 mm HF-CZT had 16×16 pads approximately 85 µm × 85 µm on a pitch of 110 µm and was biased to −1000 V with a Keithley 2410 SourceMeter, at which the dark current was approximately −0.6 µA. The ASIC was at approximately 40°C but its temperature was not explicitly monitored nor controlled. However, the ASIC and COB were powered up well before measurements commenced, so it could be reasonably assumed that their temperature had settled and was stable throughout the measurements.

#### 4.2.1. Setup on the DLS B16 Test Beamline

In CZT, charge-sharing between pixels dominates, so the use of a synchrotron was essential to get monochromatic beams of different energies and to scan pixels with sub-pixel resolution. Characterization with an X-ray beam was carried out on the B16 Test Beamline at DLS, UK (Sawhney *et al.*, 2010 ▸).

**Figure 5:**
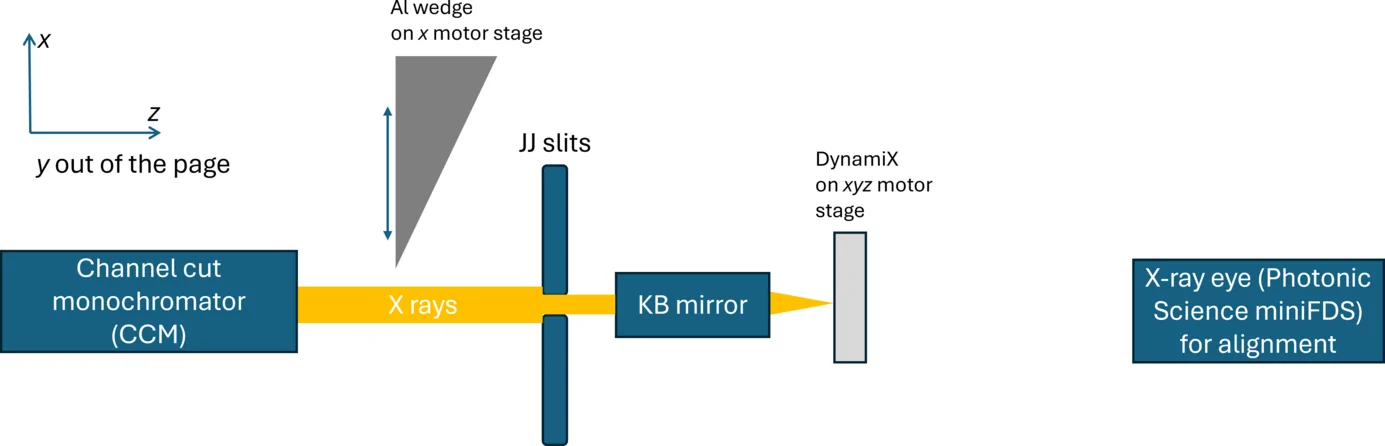
The experimental ‘CCM focused’ setup on the B16 Test Beamline at Diamond Light Source. The pinhole used for alignment is not shown, and the CCM was swapped for the DCM for later measurements.

An Si(311) channel-cut monochromator (CCM) was used with beam-defining slits and Kirkpatrick–Baez (KB) mirrors to produce a focused beam of approximately ∼60 µm × 15 µm (energy dependent). This focus size was determined using gold knife-edge scans, using step sizes of 1 µm for 15–20 µm focus sizes and 2 µm for 30–60 µm focus sizes. Beam energies of 20 keV, 30 keV, 40 keV and 50 keV were used, with the detector re-aligned to the centre of the beam with each change in energy. The *I*_0_ flux was measured using a calibrated passivated implanted planar silicon (PIPS) detector after all data had been taken. Due to the proximity of the focal plane of the KB mirror to the focusing optics flange (∼15 mm), it was not possible to measure flux with the diode and take data with the DynamiX ASIC simultaneously. A Photonic Science miniFDS camera and pinhole were used to align the beam, before DynamiX was moved into the beam path. This setup is named ‘CCM focused’ and is shown in Fig. 5 ▸. For higher-flux measurements at 20 keV, an Si(111) double-crystal monochromator (DCM) was used with both direct and focused beams. These setups are named ‘DCM focused/unfocused’, respectively.

The unfocused beam was defined with tungsten slits with sizes from 10 µm × 10 µm to 200 µm × 200 µm to irradiate sub-pixels and multiple pixels. An aluminium wedge was used to attenuate the beam (both focused and unfocused) for linearity measurements. The wedge was moved perpendicularly into the beam in 100 steps of 0.375 mm, thereby increasing the attenuation thickness by 0.375 mm × tan 15° = 0.1 mm each step. The unattenuated flux *I*_0_ and the estimated thickness of aluminium at that step number were used together to estimate the flux at each step. A total of 500000 frames were taken at each motor position.

The RF clock of the DLS electron storage ring (499.68 MHz) was taken from the beamline and used as the master clock of the ASIC, with the frame length set at 936 clocks, ensuring frames remained synchronized to one orbit of the synchrotron. However, no delay scan was carried out, so the 34 empty bunches in the DLS storage-ring fill pattern did not align with the transfer and reset time of the detector.

#### 4.2.2. Setup with laboratory-based polychromatic X-ray source

To test even closer to the design-specified flux of ∼10^11^–10^12^ photons s^−1^ mm^−2^ (20 keV equivalent), the ASIC was irradiated in an X-ray set with a tungsten target, with a tube voltage of 160 kV. The tube current was varied between 0.1 mA and 18.8 mA in steps of 1 mA, with one million frames recorded for each tube current. The calibration of DynamiX was then adjusted and the measurement repeated. As the detector head was placed as close as possible to the tube to maximize flux, manually adjustable tungsten slits were used to shield the ASIC periphery. The CZT bias current was noted from the Keithley 2410 for each X-ray tube current for each calibration. Note that the X-ray flux was not measured by an independent method – it was inferred from the DynamiX flux using the single-photon calibration outlined in Section 4.1. This setup is named ‘X-ray set (high flux)’.

**Figure 6:**
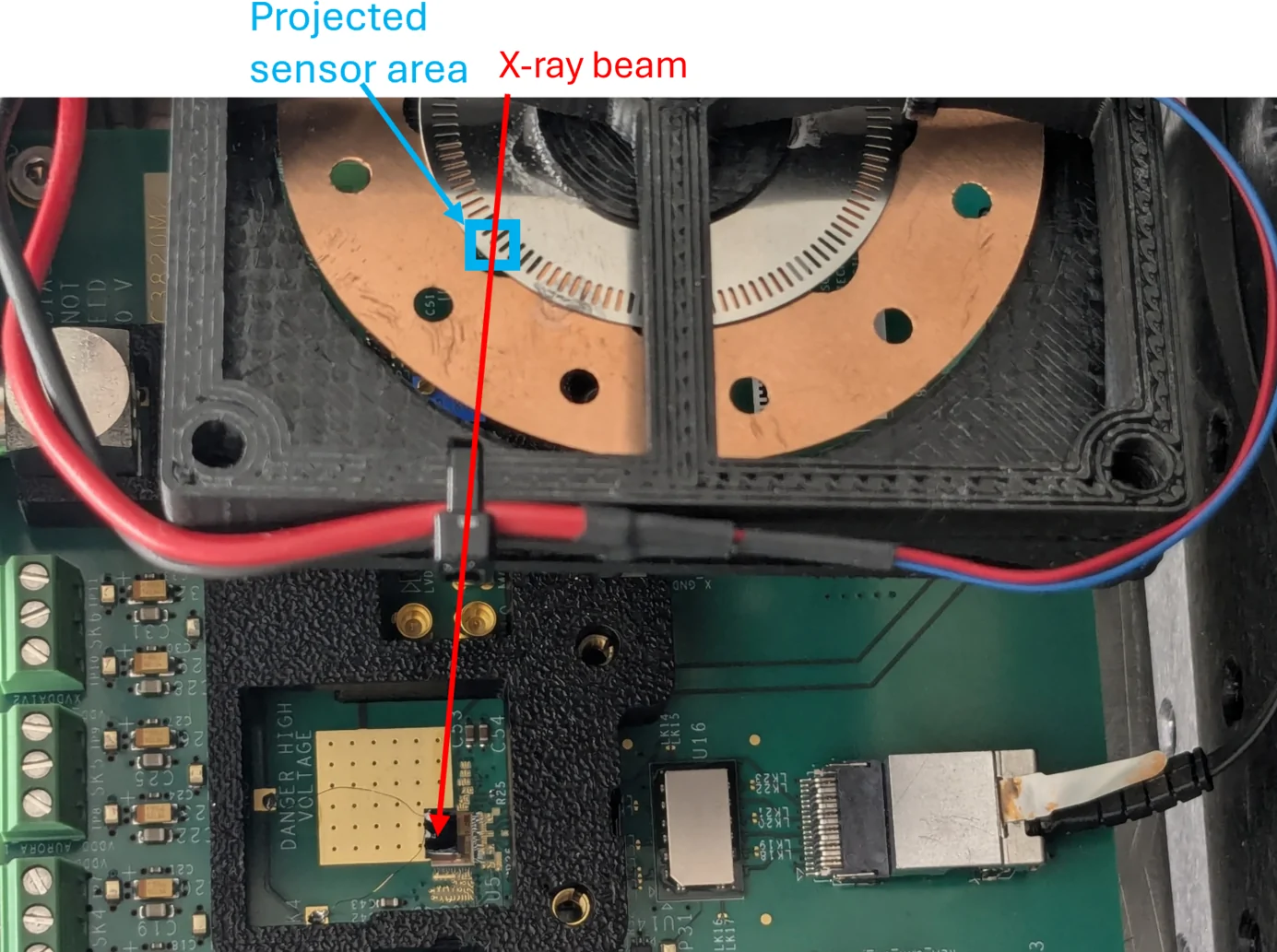
The rotating slitted disc in front of the DynamiX COB, showing the path of the X ray beam and the DynamiX sensor projected onto the disc.

To demonstrate imaging at ∼0.5 MHz frame rate, the whole 16×16 pixel array was used to image a slitted spinning steel and copper disc in a direct beam, using a tube voltage of 100 kV and current of 30 mA. This setup is named ‘X-ray set (imaging)’ and is shown in Fig. 6 ▸.

## 5. Results

### 5.1. Mismatch across the pixel array

**Figure 7:**
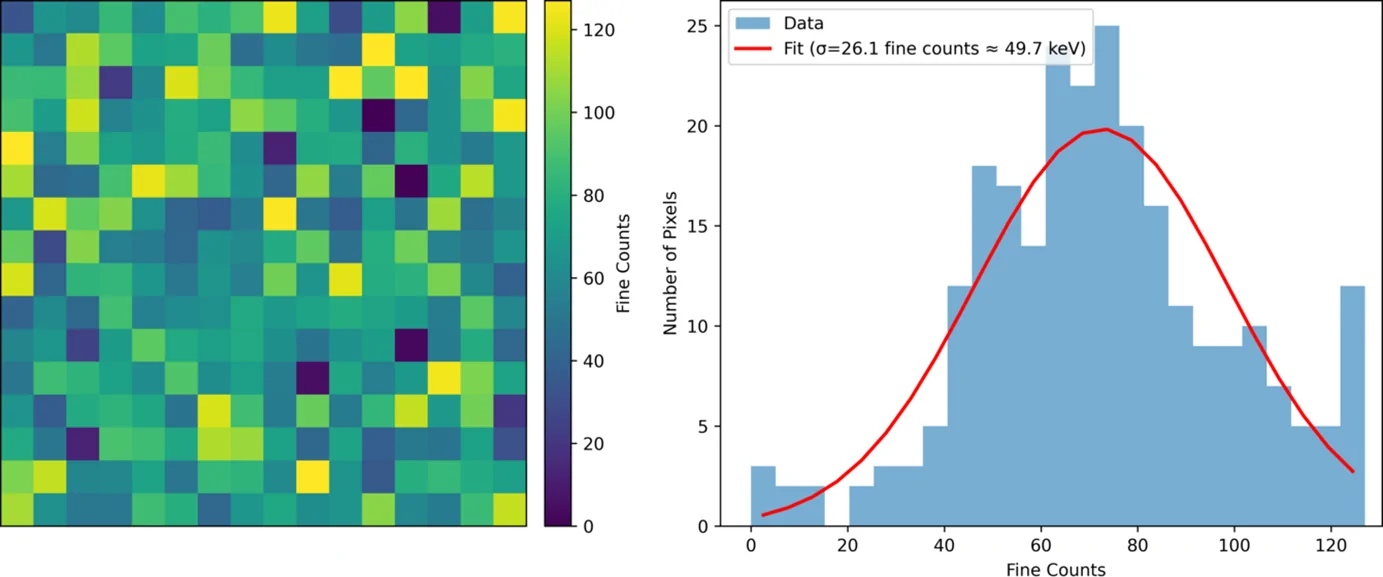
(Left) Average over 100000 frames of the array with the detector unbiased and no signal injected, to illustrate mismatch between pixels. (Right) A histogram of the average fine counts per pixel across the array, with a Gaussian fit.

No signal was injected and the detector was unbiased. The fine threshold was reduced well below the amplifier reference voltage such that all pixels were in range and counting, to best illustrate the mismatch, although note that in real use the detector is not run with the fine threshold set this low. One hundred thousand frames were taken and the fine-count average for each pixel was calculated. This is shown on the left in Fig. 7 ▸. For pixel (7,7), one fine count is ∼1.9 keV using the calibration derived in Section 5.3. These pixel means across the array are shown in a histogram, along with a Gaussian fit, on the right in Fig. 7 ▸. The measured mismatch, relative to the X-ray calibration for pixel (7,7), is σ = 49.7 keV.

The mismatch was also simulated at a schematic level, including contributions from the pre- and post-amplifiers, all current and voltage DACs, and the end-of-line buffers within the pixel cell. Note that the DAC and buffer mismatches make the simulation result pessimistic as they are globally shared across the pixel array and therefore would not have the variation used in the simulation. For the simulation, a charge of 12.5 photons of 25 keV was injected, leading to a simulated mismatch of σ = 50.0 keV, which is in good agreement with the measured value.

### 5.2. Sub-pixel raster scans

Charge sharing in CdZnTe and CdTe has been extensively studied in the literature (Veale *et al.*, 2014 ▸), and we may use models of charge-cloud properties (Koch-Mehrin, 2023 ▸) and charge sharing (Iniewski *et al.*, 2007 ▸) to estimate that around 80% of events are shared between DynamiX pixels. At X-ray energies above 25 keV, self-fluorescence of CZT, with attenuation length comparable to the pixel size (Abbene *et al.*, 2018 ▸), further contributes to charge sharing. Since the chosen calibration strategy relies on resolving photon peaks, the beam was centred within a pixel to minimize charge sharing, to understand how significantly an off-centre beam reduces the measured signal, which would degrade the spectrum quality for calibration.

**Figure 8:**
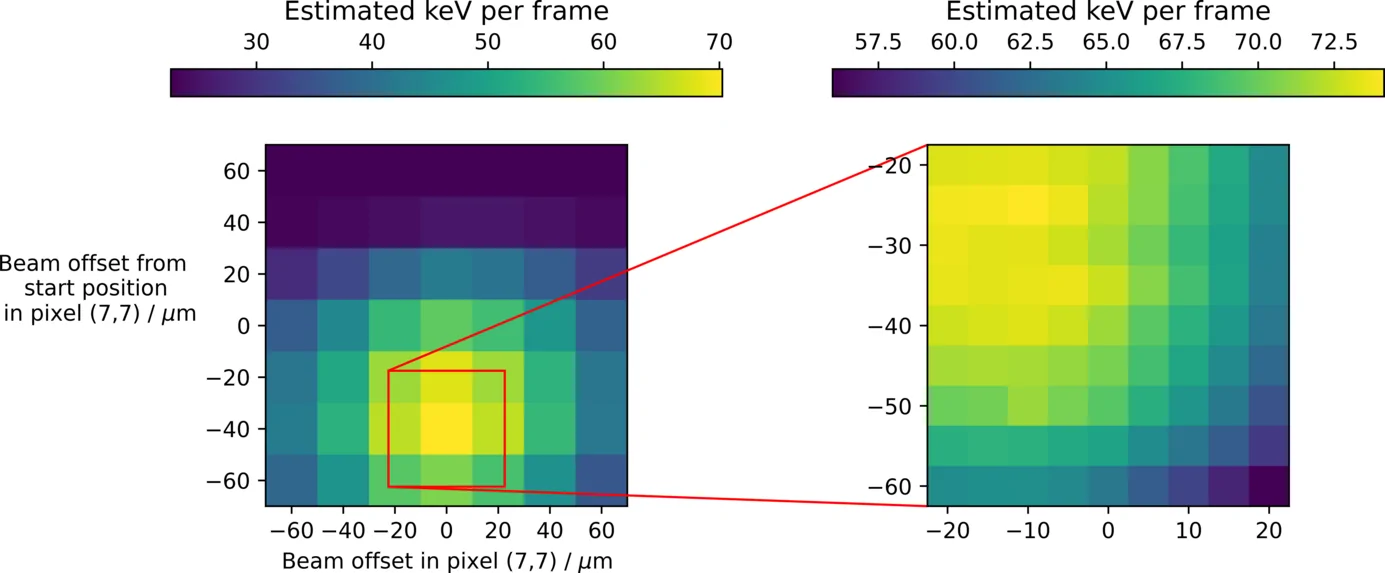
(Left) Raster scan of a pixel, in 20 µm steps, to find the centre (*i.e.* most sensitive) area of the pixel, then (right) re-centring on this ‘brightest’ motor position and raster scanning around that position in 5 µm steps.

Measurements were taken with the ‘CCM focused’ setup. To perform this scan, the 20 keV beam (along the *z* axis, with the detector in the *xy* plane) was focused in the pixel and scanned in 20 µm steps from −60 µm to 60 µm in *x* and *y*. The detector output was recorded for each position and averaged over 5 million frames. The position giving the highest DynamiX signal was set as the new origin position and a finer scan was performed in 5 µm steps over ±20 µm. Fig. 8 ▸ confirms that the (average) charge deposited in the pixel depends significantly on how well aligned the beam is to the centre of the pixel, suggesting significant charge sharing into neighbouring pixels at this pitch unless the beam is localized to the central ∼40 µm of the pixel.

Note that these measurements were taken before the pixel had been calibrated. However, since the value of the coarse threshold and the flux together meant only the fine stage was counting, it was possible to work backwards using subsequent measurements to estimate the average kiloelectronvolts deposited per pixel per frame: the offset (due to the fine threshold being too high) can be approximately corrected using the gradient in Fig. 16 and the difference between the optimum and chosen threshold in DAC codes, and the charge packet size to kiloelectronvolts ratio can be estimated from Fig. 15.

### 5.3. Calibration of the fine stage

#### 5.3.1. Setting the charge-cancellation packet size

**Figure 9:**
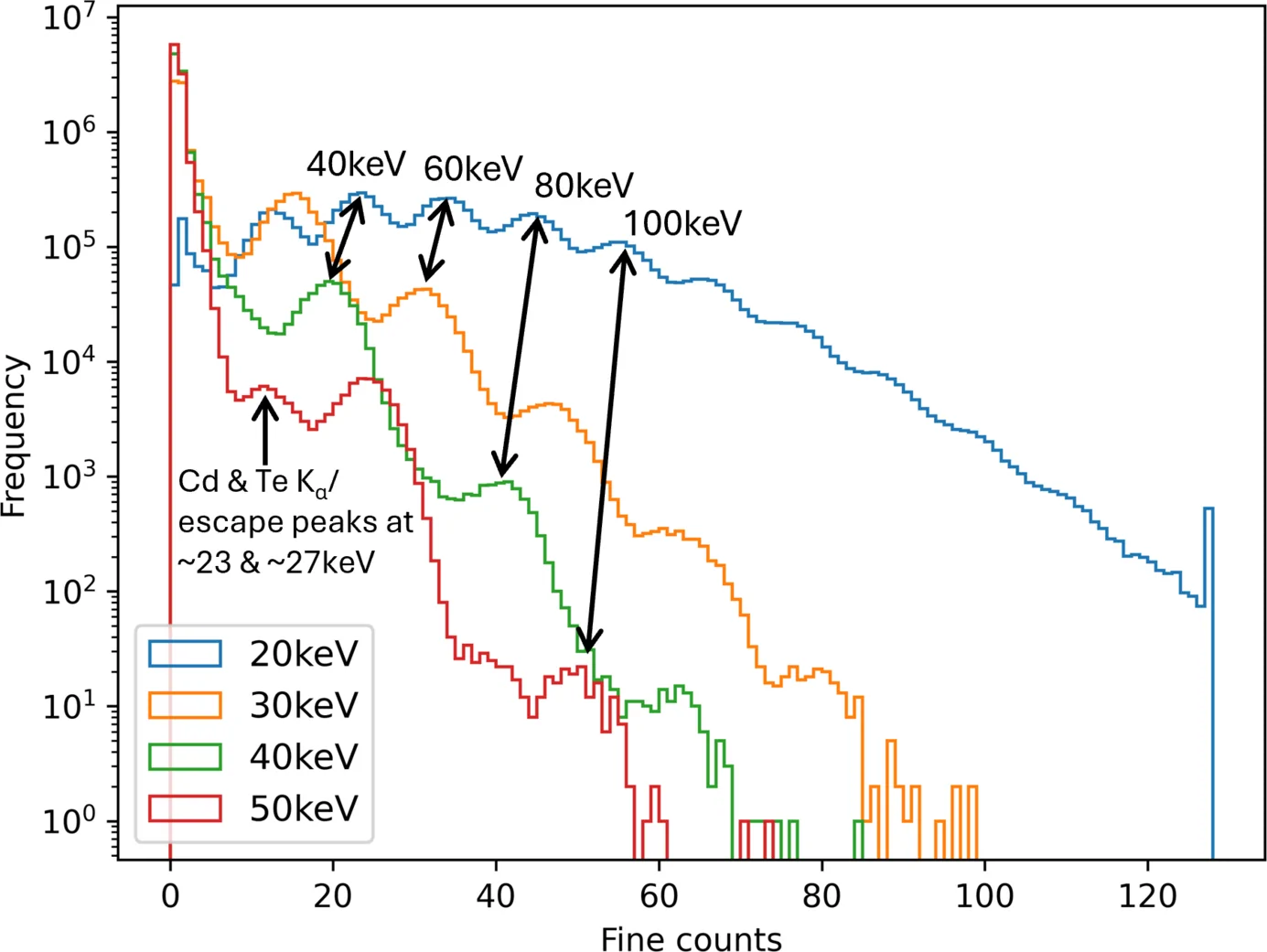
Histograms of 10 million frames for one pixel irradiated with different energies, showing *N*-photon peaks, and the cadmium and tellurium fluorescence and escape peaks.

A histogram of fine-count values for 10 million frames for different X-ray energies is shown in Fig. 9 ▸, where the nominal charge packet size was approximately 2 keV. Unless otherwise stated, measurements in this subsection were taken with the ‘CCM focused’ setup.

The horizontal offset between peaks of equivalent energy in different beam energy series may be due to effects in the HF-CdZnTe sensors. Previous measurements on these devices with other ASICs (Cline *et al.*, 2024 ▸) have demonstrated the presence of an ‘excess leakage current effect’. At higher X-ray fluxes, it is hypothesized that trapping at the metal–semiconductor interface leads to a lowering of the potential barrier, allowing more thermally-generated charge to flow out of the detector. In integrating detectors, this manifests as a shifting of the pixel pedestals under irradiation relative to the dark pedestal level.

For the beamline setup used during these measurements, there are significant reductions in the photon fluxes at higher energies, with the (focused) flux delivered to the pixel decreasing from 1.6 × 10^6^ photons s^−1^ at 20 keV to 7.8 × 10^4^ photons s^−1^ at 50 keV as measured by the calibrated PIPS photodiode. As a result, a shift in the pixel pedestal is observed when compared with the higher energy.

**Figure 10:**
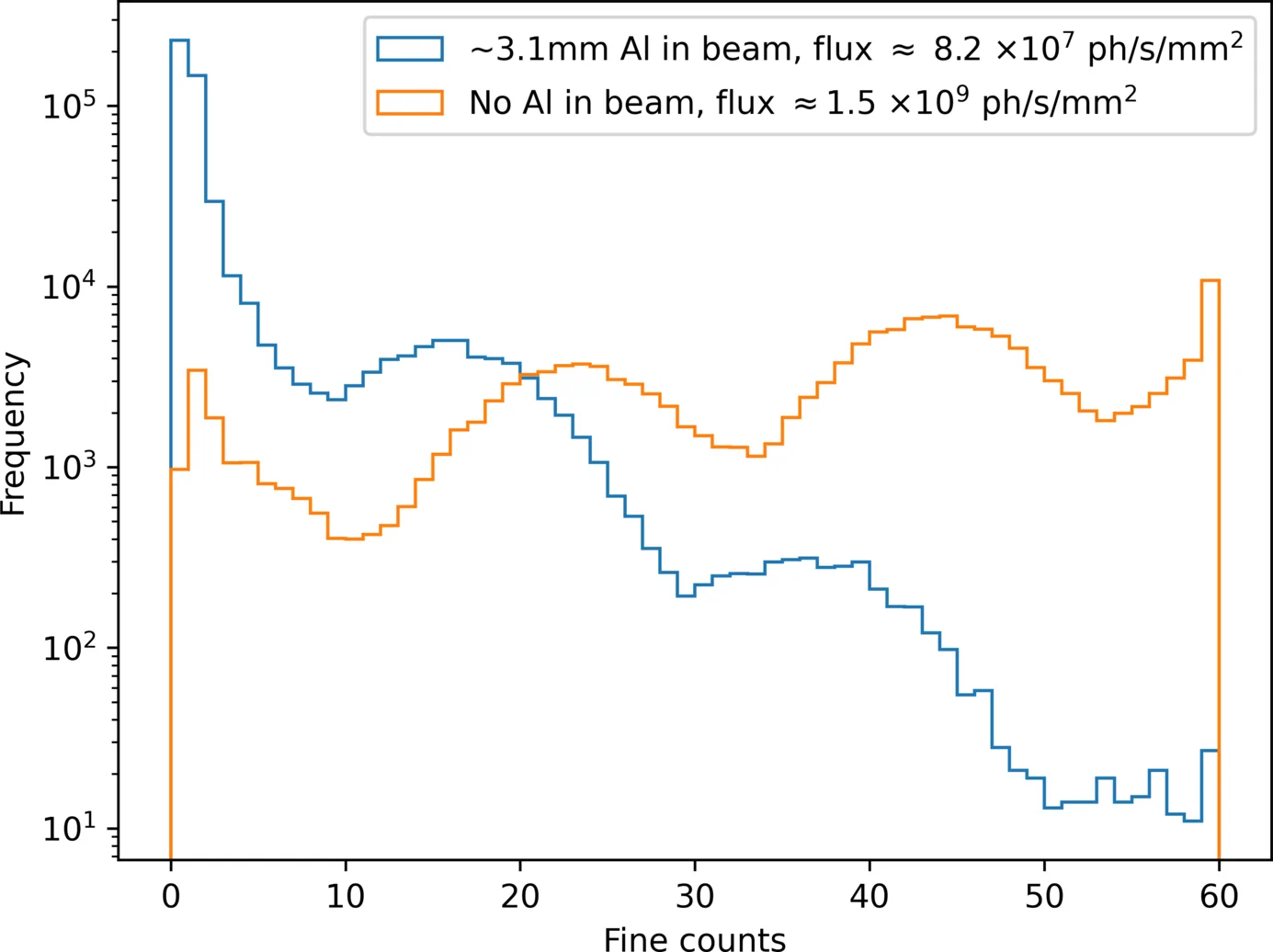
Histograms of 10 million frames of pixel (7,7) under two different fluxes of 20 keV beam, illustrating a shift in the baseline.

This effect can also be seen at one energy, by changing the flux on the detector by increasing the attenuation. Fig. 10 ▸ shows how the one- and two-photon peaks with the 20 keV beam shift to higher fine-count values at higher fluxes. These measurements were taken with the ‘DCM unfocused’ setup.

The shift in the one-photon peak is approximately 7.5 fine counts, with the two-photon peak shifted by 8.3 fine counts. Since the spacing of the one- and two-photon peaks for each flux is approximately 20 fine counts, we infer a flux-induced shift of 7–8 keV. Using the frame length of 1.873 µs, the beam area of 50 µm × 50 µm and the electron–hole creation energy of 4.6 eV in CZT, we can estimate a flux-induced excess leakage current density of ∼50–60 nA mm^−2^ in this flux range. Note that this value is more than an order of magnitude greater than the value measured by Cline *et al.* (2025 ▸), although it should be noted that their value was measured at significantly lower flux. More work is needed to gain a full understanding and characterization of this effect with the DynamiX detector.

**Figure 11:**
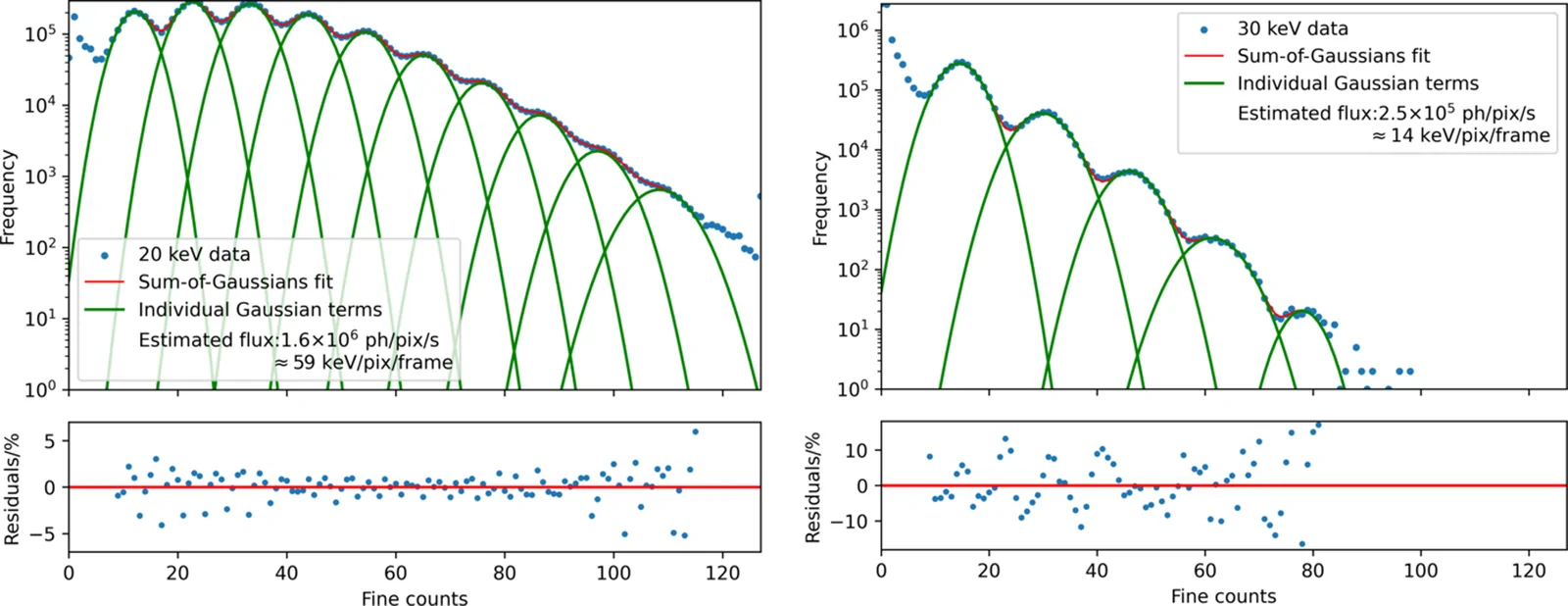
Sum-of-Gaussians fits to histograms of (left) 20 keV and (right) 30 keV photon peaks from a single pixel, allowing an estimation of the average kiloelectronvolts deposited per frame in the pixel (given in the insets). Only points shown in the residuals contributed to the fits.

**Figure 12:**
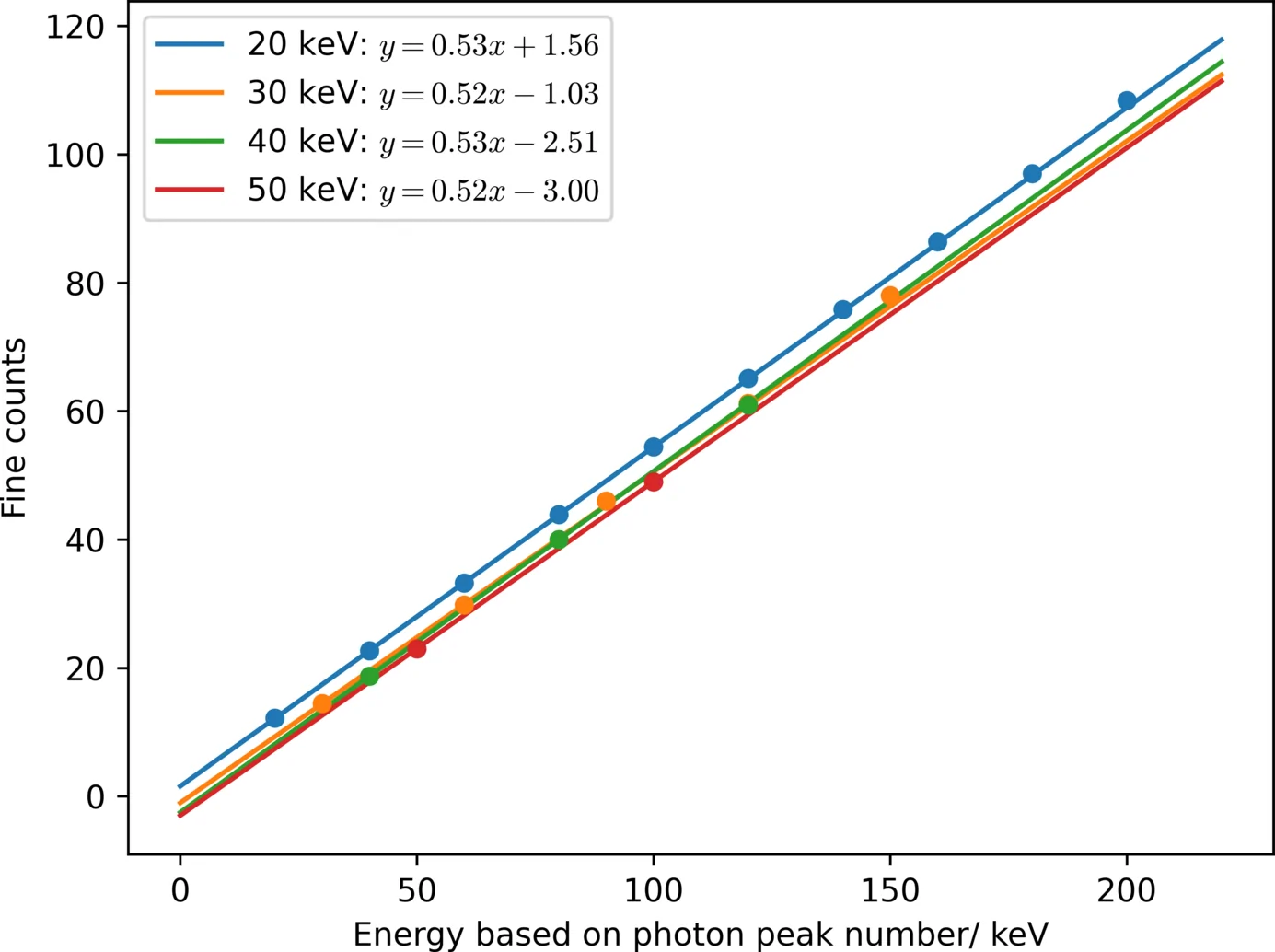
The positions of the *N*-photon peaks for different energies of the beam, for a single pixel. The offset dependence with energy is attributed to sensor effects induced by the different beamline fluxes.

To understand the performance of the detector across different beam energies, a sum-of-Gaussians function was fitted to the histograms in Fig. 9 ▸, and these are shown for 20 keV and 30 keV in Fig. 11 ▸. The fit quality (not shown) for the 40 keV and 50 keV data is lower, and this is thought to be due to the escape peaks from cadmium and tellurium. The fine-count value for the centroids of each *N*-photon peak, for each energy series, is shown in Fig. 12 ▸, with weighted least-squares linear fits. The average flux, and hence kiloelectronvolts deposited per frame, was estimated from the histograms in Fig. 11 ▸, by first using the intercept from Fig. 12 ▸ to correct for the shift due to the excess leakage current, then calculating the total number of fine counts in the histogram. Finally, the gradients from Fig. 12 ▸ were used to convert the total fine counts to kiloelectronvolts. The estimated values are in good agreement with the independently measured values: for 20 keV, the measured and estimated fluxes agree to two significant figures, and to one significant figure at 30 keV. Only the fine-count values were used to estimate the flux. While the histogram has overflowed into the coarse stage for the 20 keV beam measurements, shown by the sudden rise at 127 fine counts, this was the case only for less than 1000 frames out of the 10 million frames recorded, so has negligible impact on a flux estimation.

The gradient of each line in Fig. 12 ▸ is similar, implying that the fine count to kiloelectronvolts conversion is not dependent on X-ray energy. However, the intercepts are significantly different, supporting the idea that the shift is due to an offset effect in the CZT at higher fluxes, and indeed the shift is correlated with the amount of charge being deposited (*i.e.* the flux).

The digitization resolution (the gradient, *i.e.* the fine count to kiloelectronvolts conversion) is modified by changing the charge packet size. As the fine counter is fixed at 7 bits, a higher resolution (less energy per bin) can be obtained at the expense of dynamic range. For calibration, a small charge packet size is used, but in real-world use a larger packet size of 0.25, 0.5 or 1 photon per fine count would be used to obtain maximum counts. Simulation suggests the maximum coarse packet size is around 80 photons at 25 keV.

**Figure 13:**
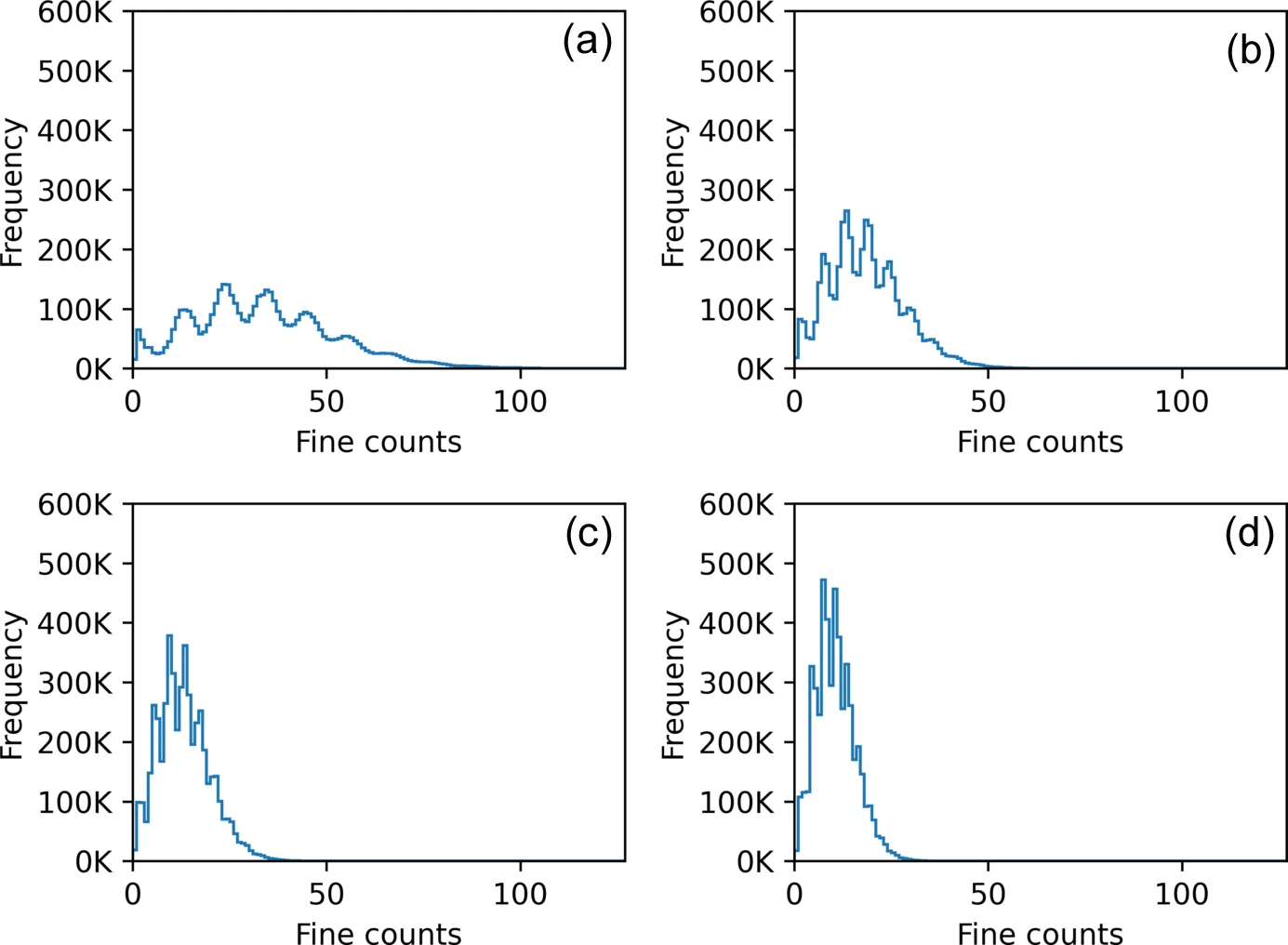
Histograms of the fine-stage output of a single pixel over 10 million frames for increasing fine-stage charge cancellation packet size from panel (*a*) to panel (*d*), under constant irradiation.

Fig. 13 ▸ shows the effects of changing charge packet size on the measured spectrum at 20 keV at a fixed (focused) flux of 1.6 × 10^6^ photons s^−1^ into the pixel. As the charge packet size is increased, fewer packets are needed to remove the same charge, so the peaks become narrower and move to the left.

**Figure 14:**
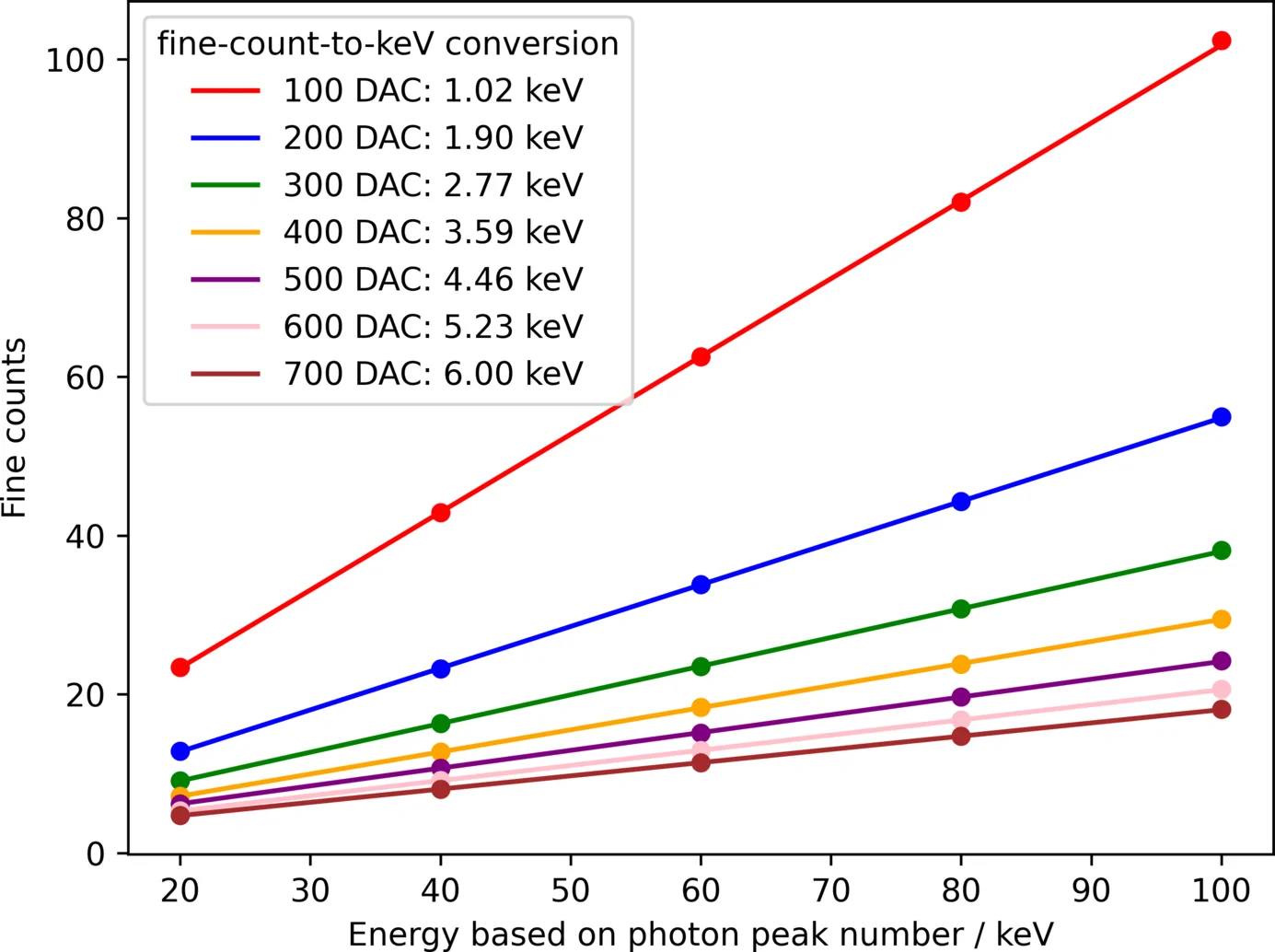
The change in the photon peak positions as the fine-stage charge packet size (DAC code) is changed. Each series represents a different charge packet size, and therefore a different fine count to kiloelectronvolts conversion.

A sum-of-Gaussians function was fitted to these photon peaks, and the peak positions are plotted for each charge packet size in Fig. 14 ▸. The fine count to kiloelectronvolts conversion for each charge packet size is given by the gradient of the weighted least-squares fit, which used the fit uncertainties of the centroids of the photon peaks.

**Figure 15:**
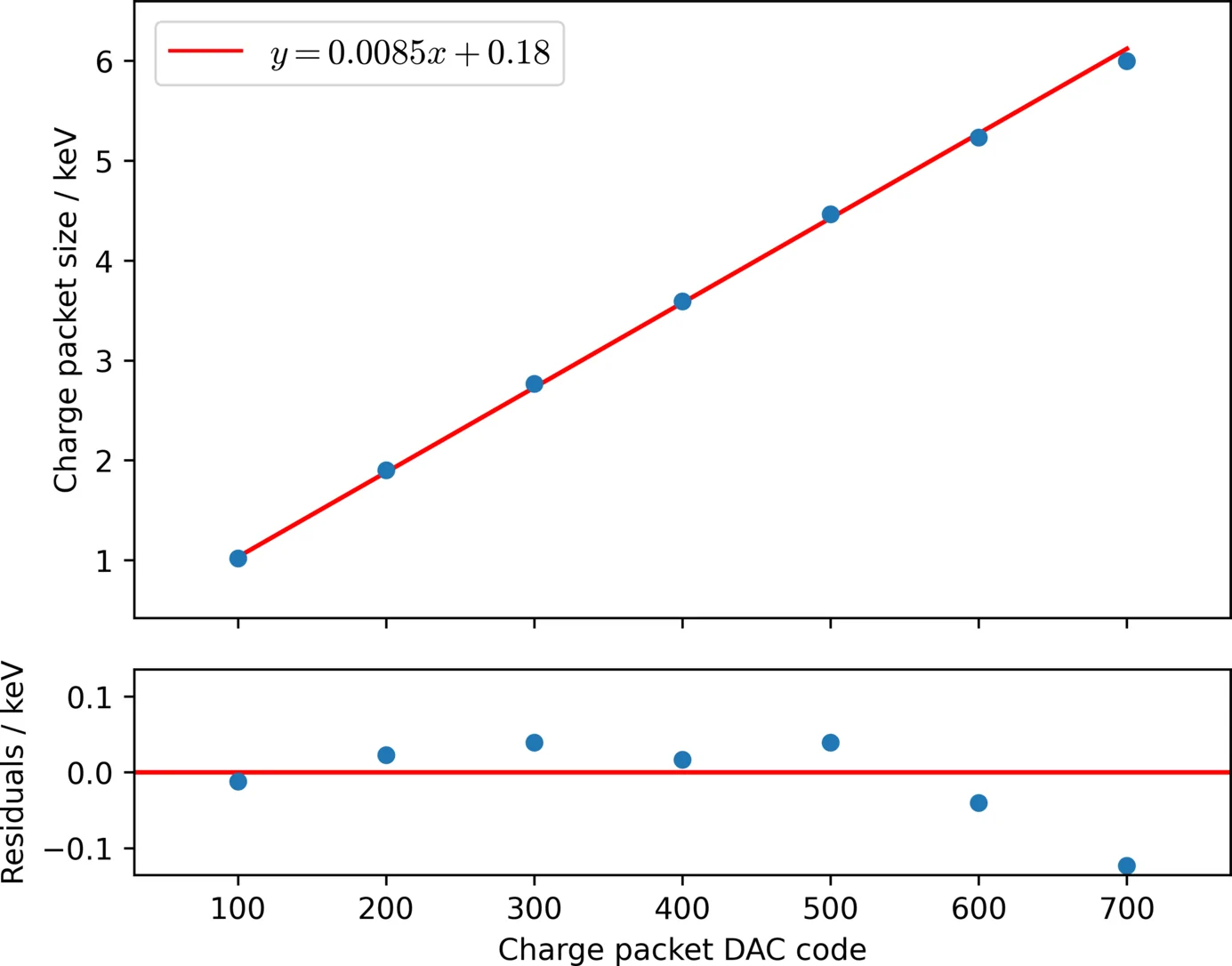
The linearity of the charge packet size as a function of its DAC code, for the fine packet.

In Fig. 15 ▸, these gradients are plotted against their respective DAC code setting for the fine-stage charge cancellation packet size, allowing a determination of the linearity of the current DAC and current mirror to the pixel. Excellent linearity is seen over the DAC range 100 to 700, corresponding to packets ∼1 keV → ∼6 keV. This represents about one sixth of the available range for this current DAC.

#### 5.3.2. Fine-stage threshold calibration

**Figure 16:**
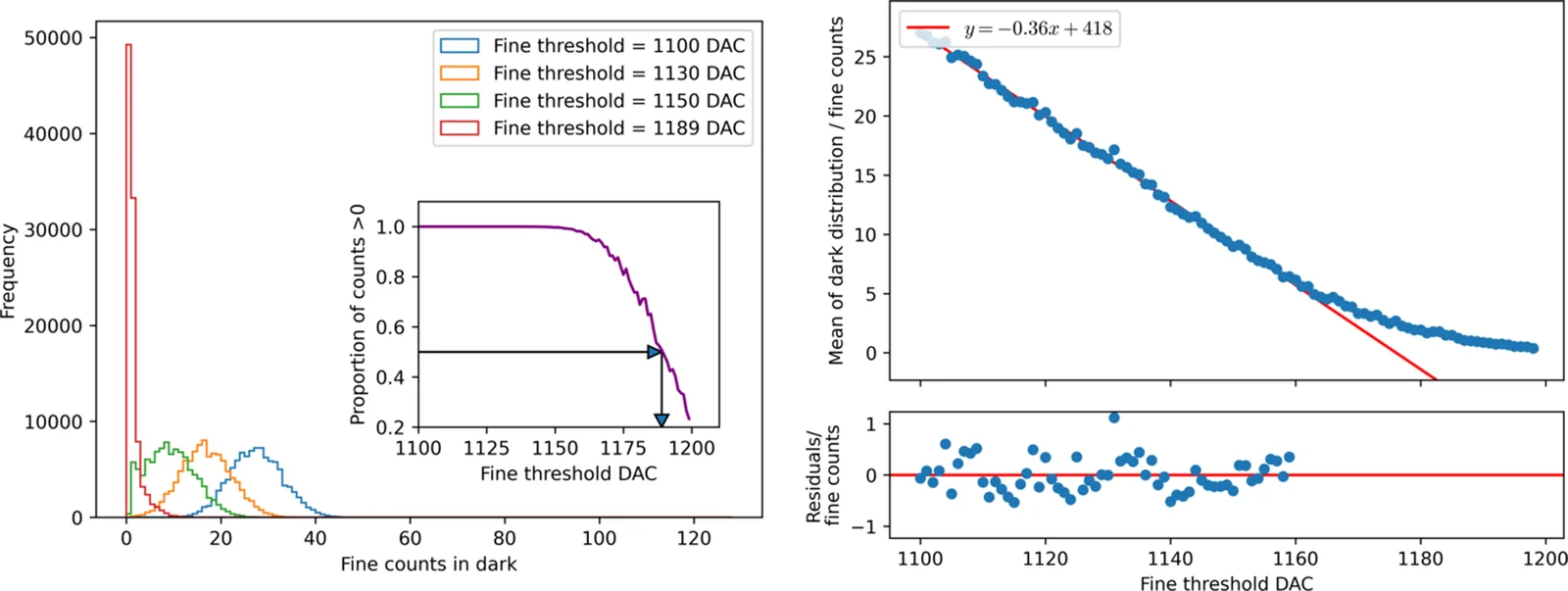
(Left) Histograms of 100000 frames in the dark with different fine thresholds, for a single pixel. (Left inset) To centre the noise peak on approximately zero fine counts, the threshold at which 50% of frames give counts greater than zero is chosen, corresponding to a fine threshold of 1189. (Right) The means of the dark distribution for different fine thresholds (only those points shown in the residuals contributed to the linear fit).

Once the fine count to kiloelectronvolts conversion had been determined from the *N*-photon peak spacing, the fine threshold was adjusted in the dark to centre the noise peak approximately on zero fine counts. This is approximately equivalent to 50% of frames giving counts greater than zero, which is the case for DAC code = 1189. As the threshold is increased, the count values become smaller, since the difference between the reference and the threshold decreases. This is shown in Fig. 16 ▸.

Fig. 16 ▸ shows that the width of the 1189 distribution is less than half of the width of *e.g.* the 1100 distribution, as one might initially have expected if the 1100 Gaussian is simply clipped at zero. We hypothesize that this is due to the sampling of two different noise regimes – when the fine count values are large, the noise on the charge packets is the dominant source, but as the number of cancellations reduces, the (lower) noise on the comparator threshold becomes the dominant source.

Once a fine count to kiloelectronvolts estimate has been obtained (Fig. 14 ▸), the noise performance can be estimated from the width of a Gaussian fitted to the histogram of the dark pixel output when the noise distribution is well away from zero (*e.g.* fine threshold = 1100 DAC, the blue histogram in Fig. 16 ▸), and therefore not skewed. Using a fine packet size of ∼1 keV, this yields σ = 5.6 ± 0.1 fine counts, corresponding to 5.7 ± 0.1 keV, leading to a root-mean-square (r.m.s.) noise of around 1200 e^−^ using the electron–hole creation energy of 4.6 eV in CZT. The design specification was that a typical fine packet equals 0.2 photons at 30 keV energy. During the ASIC design phase, the noise performance of the fine stage was simulated by injecting 12.5 photons of 30 keV and passing these through the coarse stage by disabling coarse cancel clocking. The standard deviation was around 0.17 photons, leading to an r.m.s. of around 1100 e^−^, which is in reasonable agreement with the measured value. Note that this simulation did not take account of system-level noise sources such as the power supply.

To calibrate the fine threshold for larger fine packet sizes, for which the dark distributions would be narrower, an alternative method of setting the fine-stage threshold may be necessary, for example by using the test pulse to inject one fine packet’s worth of charge and then sweeping the fine threshold. However, the test pulse circuitry on DynamiX was not optimized for very low-charge injection, so further work is needed to characterize this.

### 5.4. Coarse-stage calibration

Once the fine stage has been calibrated, the coarse stage is calibrated using the overlap in dynamic range between the coarse and fine stages (with 1 coarse count = 64 fine counts, the fine stage can measure up to 2 coarse counts).

#### 5.4.1. Coarse threshold calibration

**Figure 17:**
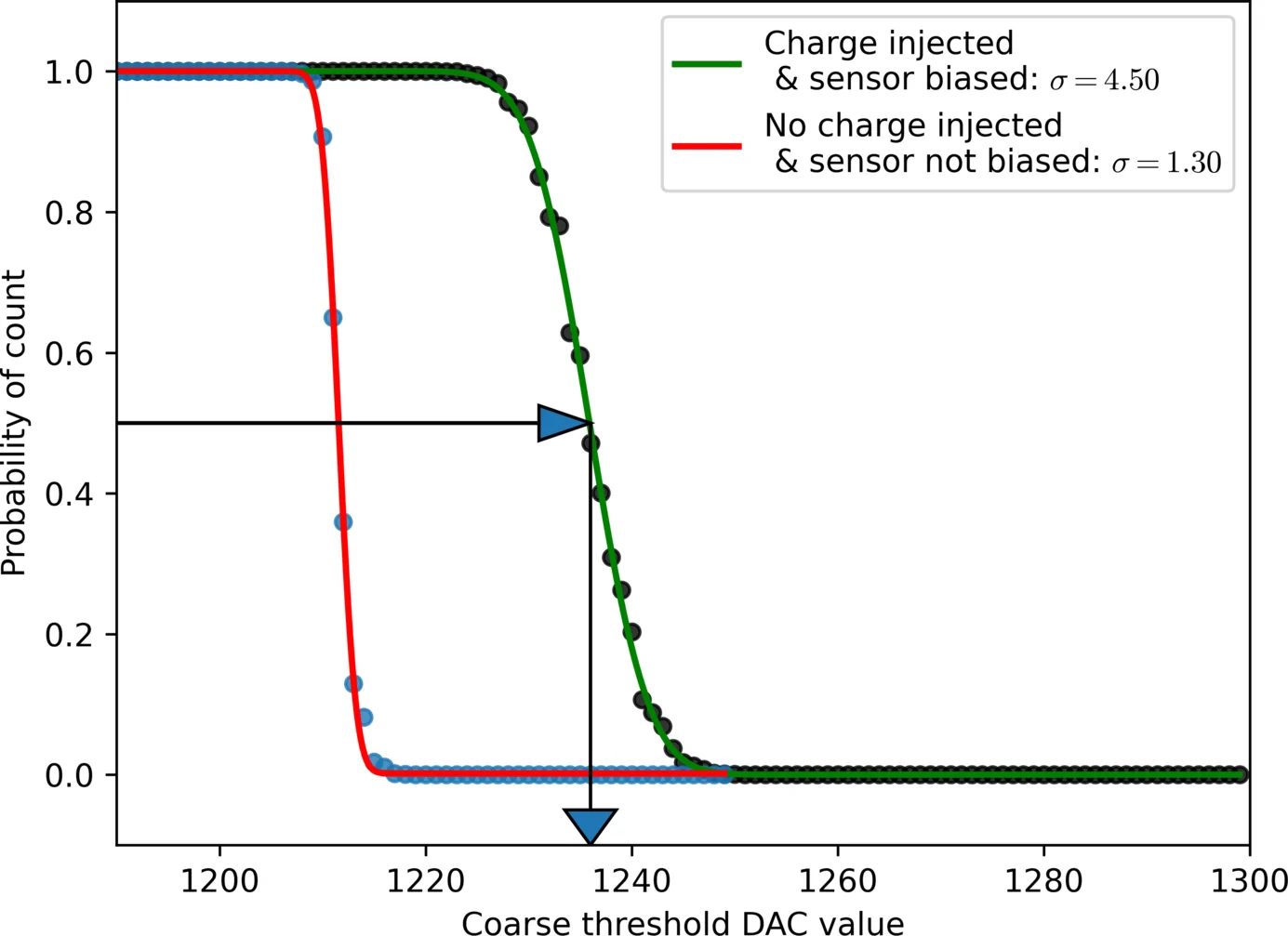
The coarse threshold is swept in steps of one DAC code while injecting 64 fine counts = 1 coarse count, to find the value which gives a 50% probability of triggering for this pixel. Data points are averaged over 10000 frames. A similar threshold sweep with no charge injection and no sensor bias is shown for comparison.

The coarse stage should count once if charge equivalent to 64 fine counts is input. With the coarse stage still ‘disabled’, a test pulse duration and current (around 110 nA for 20 ns) were found which gave 64 fine counts in the (calibrated) fine stage. The threshold of the coarse stage was then swept to find the threshold which corresponds to a charge of 64 fine counts = 1 target coarse count. This ‘S curve’, with each data point averaged over 10000 frames, is shown in Fig. 17 ▸. The threshold is set a little higher to prevent possible over-cancellation below the baseline.

#### 5.4.2. Coarse cancellation packet size calibration

**Figure 18:**
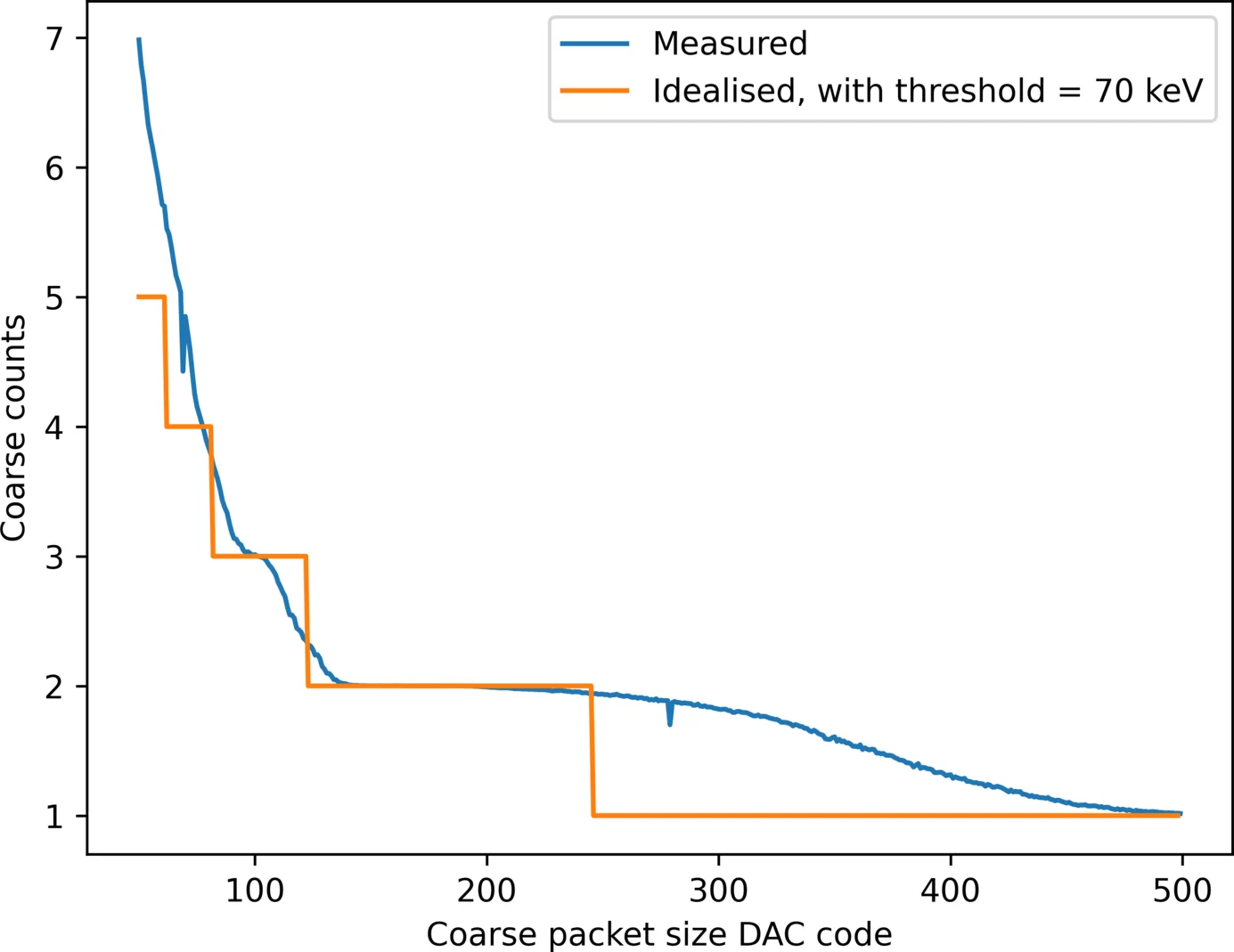
Injecting three coarse counts’ worth of charge and sweeping the coarse cancellation packet size in steps of one DAC. Each data point is the average of 10000 frames.

Charge equivalent to three coarse packets was injected into the coarse stage (with no X-ray signal) by tripling the duration of the test pulse used for the coarse-stage threshold calibration in Fig. 17 ▸, corresponding to 110 nA applied for 60 ns. The packet size was then increased and the number of cancellations (*i.e.* coarse count value) was measured. The idealized behaviour in a noise-free system with a perfectly linear coarse cancellation packet size DAC and threshold of 70 keV is shown alongside the measured result in Fig. 18 ▸. Note that the measured result includes contributions of noise from the comparator, the preamplifier, the voltage and current DACs, and timing jitter, and hence the sharp steps in the ‘simulated’ plot are in fact measured as ‘S curves’, as in Fig. 17 ▸. It is hypothesized that the S curves become wider for fewer counts (higher cancellation currents) because the error on the charge packet increases as the charge increases for constant jitter. The maximum packet size corresponding to two coarse counts (not three, since the threshold is greater than the charge packet size) is chosen. Since this value is not clear due to the noise in Fig. 18 ▸ on the S curve between counts 2 and 3, the midpoint of the S curve is chosen, and this is then subsequently further fine tuned with the test pulse in the following subsection.

#### 5.4.3. Verifying the calibration

The total equivalent fine-count value *T* for a frame is given by
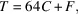
where *C* is the coarse counter value and *F* is the fine counter value for that frame. A correct calibration ensures that the charge cancelled by one coarse count is equal to that cancelled by 64 fine counts.

**Figure 19:**
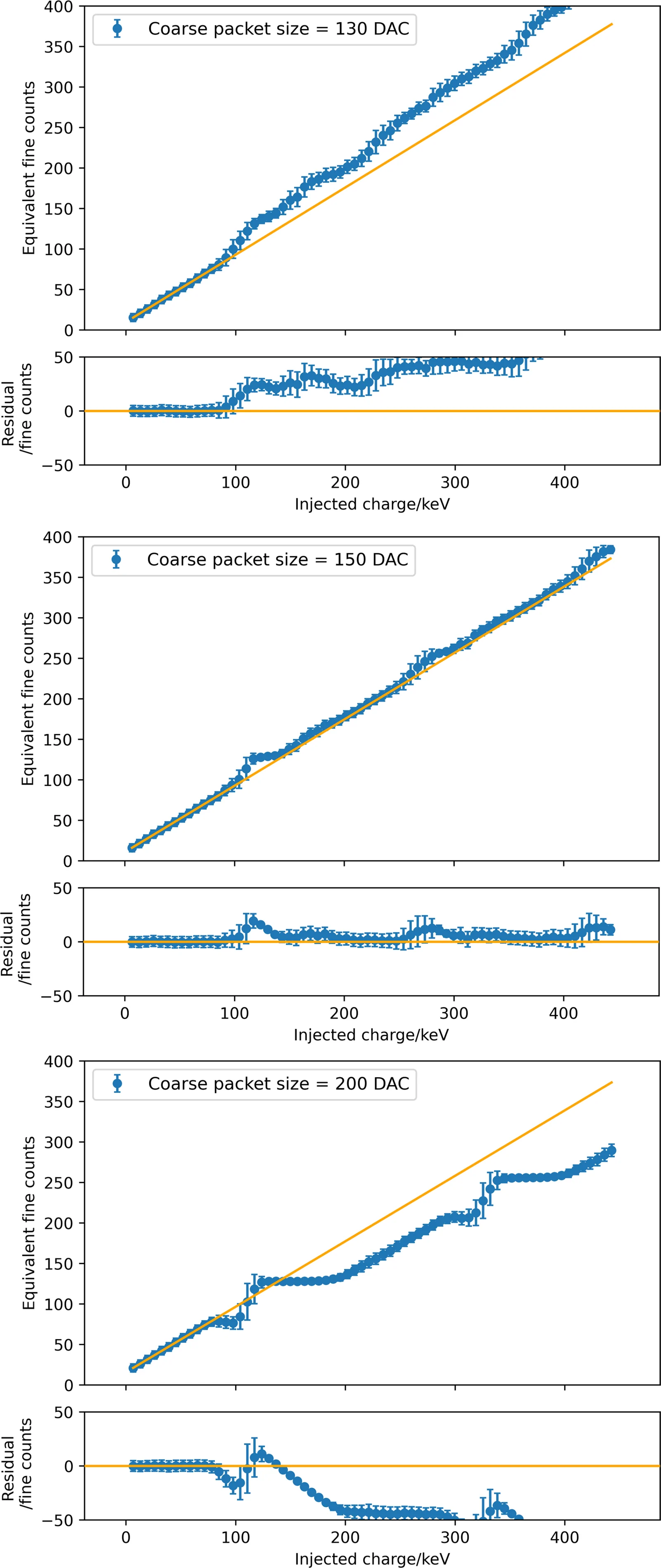
Quality of calibration when the coarse cancellation packet size DAC code is (top) too small, (middle) close to optimum and (bottom) too large. Each point is the average of 10000 frames and the error bars are the standard deviations of the equivalent fine count across those 10000 frames.

To verify the calibration, the test pulse *current* is kept constant while the pulse *duration* is slowly increased. For small values of injected charge, only the fine stage will count. These determine the ‘correct’ gradient as given by the orange lines in Fig. 19 ▸.

Fig. 19 ▸ shows the effect of varying the coarse cancellation packet size. If the packet is too small, less charge than needed is removed by one coarse packet, causing the total equivalent fine counts to overshoot. If the packet is too large, too much charge is removed by one coarse packet, and hence the total equivalent fine count undershoots. For optimal calibration, the charge removed by 1 coarse packet should equal 64 fine counts, and the fit remains good. The optimal calibration is therefore achieved by minimizing the deviation from the orange line, *i.e.* minimizing the sum of the (absolute) residuals. Periodic deviations are seen when a coarse count is recorded, even though subsequent data points are well-aligned to the target calibration in orange. We note that the error increases once the coarse threshold is exceeded, and that, for the close to optimum calibration, the error is higher for injected charges just *below* the midpoint of the deviation, but not those injected charges just *above* it, suggesting a residual misalignment between the coarse threshold and packet size.

### 5.5. Linearity performance up to ∼900 photons per pixel per frame (equivalent at 20 keV)

**Figure 20:**
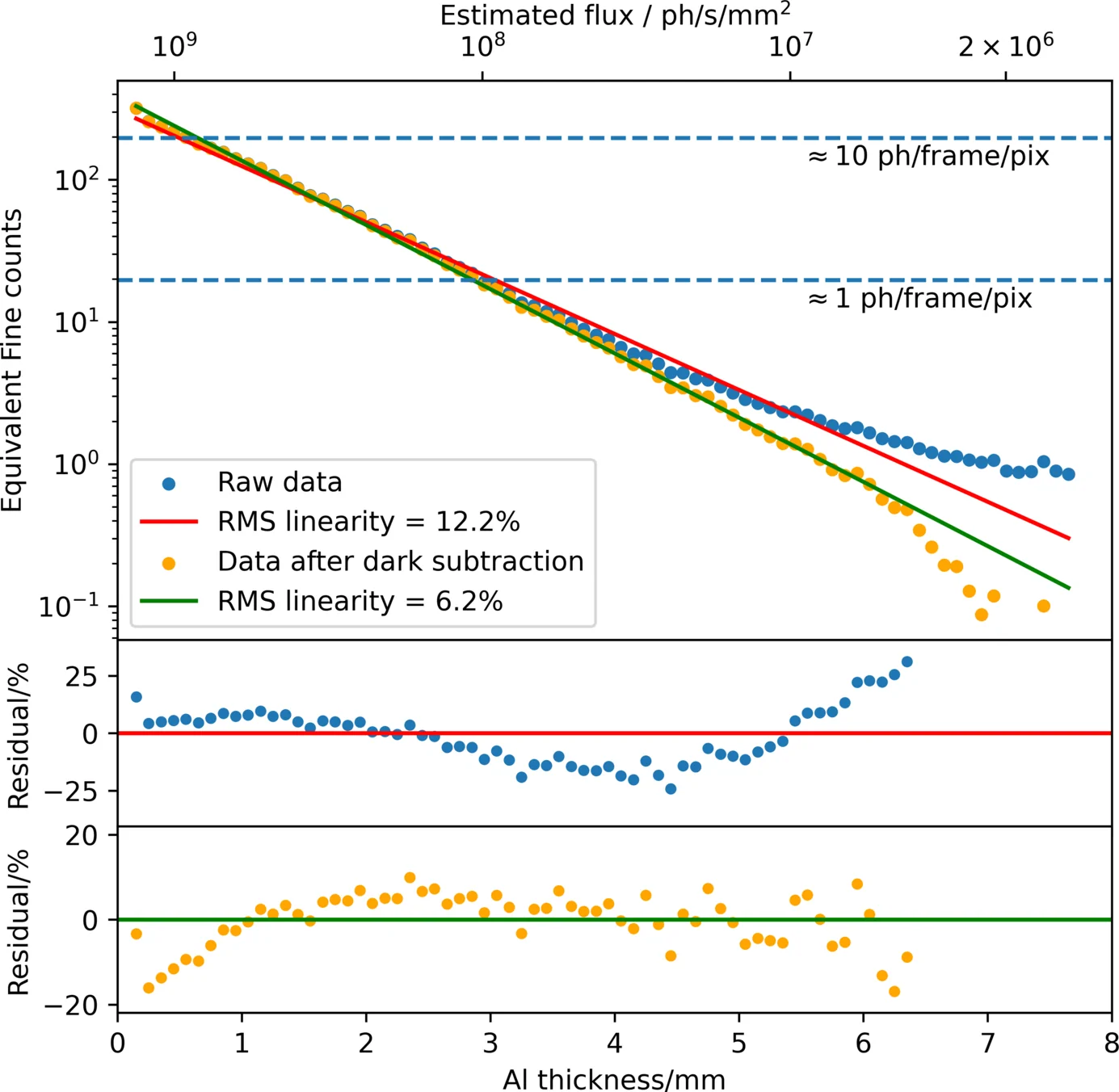
Varying the flux of a 20 keV beam with an aluminium wedge and recording the total equivalent fine counts in a pixel, averaged over 500000 frames, as a function of thickness. The horizontal estimates of 1 and 10 photons per pixel per frame are based on the calibrations in Section 5.3, but note that no correction has been made here for the ‘excess leakage current’ offset. Only the points shown in the residuals contributed to the linear fits and the r.m.s. linearity calculation.

The ‘DCM unfocused’ setup was used for these measurements, since this gave the highest available flux on the B16 Test Beamline. A total of 500000 frames were taken in the dark and at each aluminium wedge attenuator position, and the mean total equivalent fine counts per frame was calculated. The mean ‘dark level’ was calculated to be around 1 fine count, as seen in the red histogram in Fig. 16 ▸ (not zero, since half of the frames give counts > 0). Fig. 20 ▸ shows both the uncorrected raw data and the data with the mean dark counts per frame (*i.e.* ∼1 fine count) subtracted. Since the mean of the dark distribution is close to 0, while this makes little difference to the percentage deviation when the total equivalent fine counts *T* (*i.e.* flux) is large, for very low fluxes this has a large impact. Therefore, subtracting the dark mean almost halves the r.m.s. of the residuals from 12.2% to 6.2% in the flux range ∼10^6^ photons s^−1^ mm^−2^ to ∼10^9^ photons s^−1^ mm^−2^ (Fig. 20 ▸). Note that this method of assessing linearity is significantly dependent on the uniformity of the wedge and the quality of the alignment of the wedge axis to the beam, so this may represent an underestimate of the linearity performance of DynamiX. Furthermore, at higher attenuator thicknesses, the relative contribution of the third and fourth harmonics to the beam increases, and the flux estimation becomes less reliable. Note also that the periodic deviation features seen in Fig. 19 ▸ are not present, which is thought to be because each frame is dominated by Poisson statistics and those effects are averaged out.

**Figure 21:**
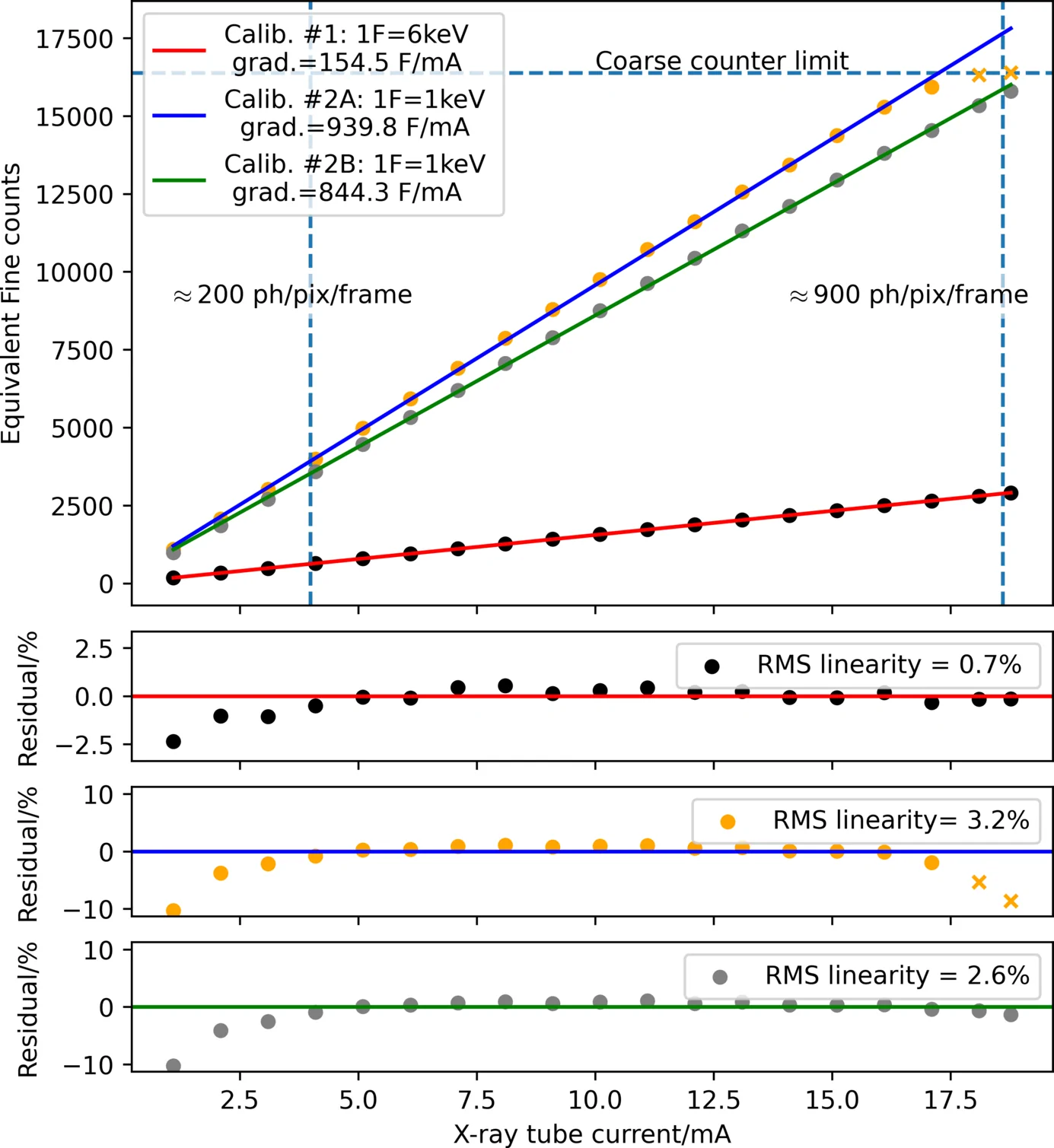
Varying the flux (but not the spectrum) from an X-ray set by varying the tube current, for three different calibrations of pixel (7,7). As the fine count to kiloelectronvolts conversion is now series-dependent, the approximate fluxes corresponding to ∼200 photons per pixel per frame and ∼900 photons per pixel per frame are shown as vertical lines instead. Data points with a cross marker were not included in the linear fit. The coarse counter limit is at (2^8^ − 1) × 64*F* (fine counts).

**Figure 22:**
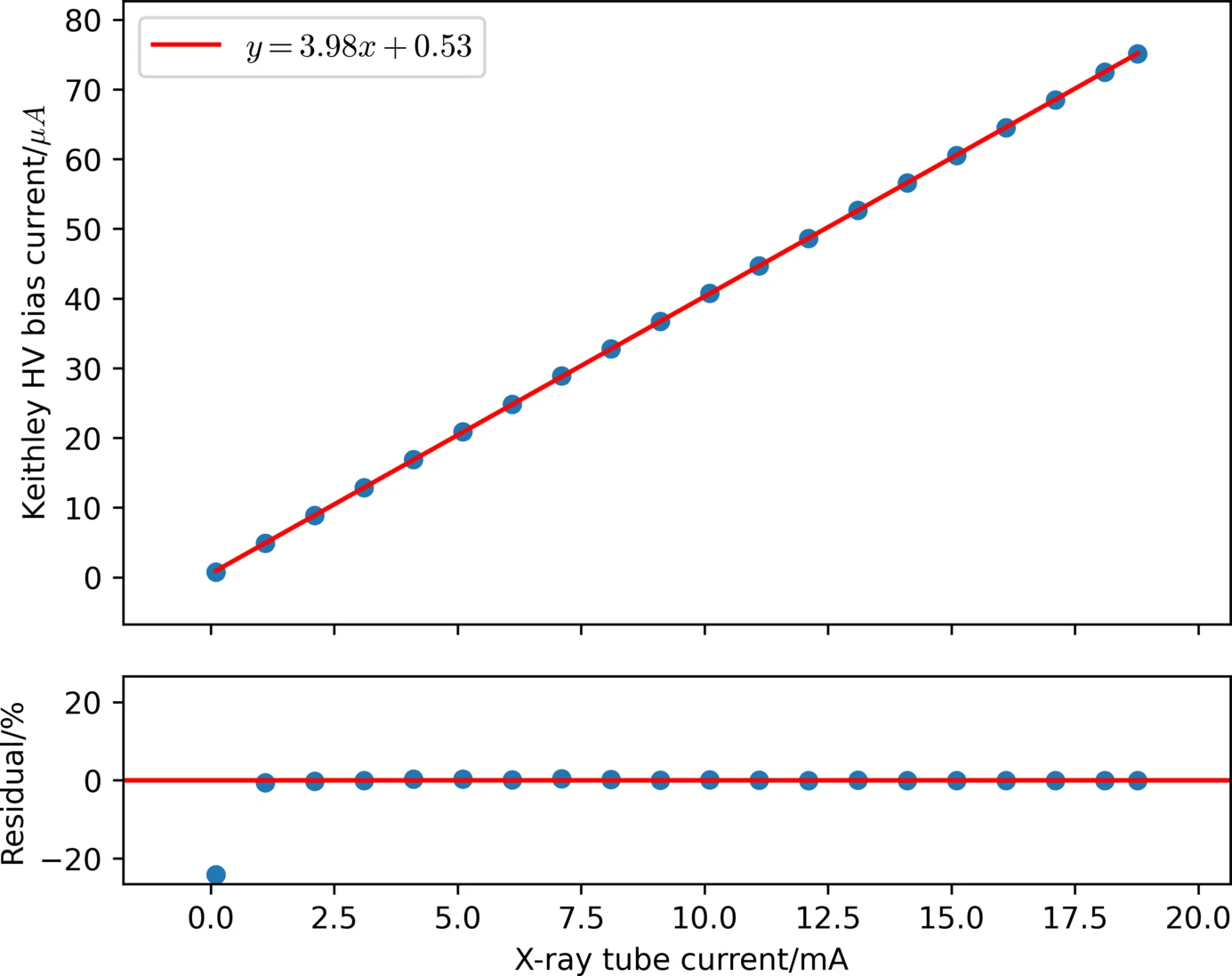
Verifying the linearity of the X-ray tube output with tube current, by recording the bias current supplied by the Keithley to the detector.

Results from the ‘X ray set (high flux)’ setup are shown in Fig. 21 ▸. To verify that the output flux of the X-ray tube itself increased linearly with tube current, the HV bias current supplied by the Keithley to the CZT on DynamiX is plotted against the X-ray tube current in Fig. 22 ▸. A Keithley bias current measurement was taken for each tube current for each calibration setting, and each data point is the average of the three measurements. Since the residuals suggest a significant percentage (though not absolute) deviation from flux versus tube current linearity at the lowest tube current of 0.1 mA, this data point is not included in the analysis in Fig. 21 ▸.

It was possible to reach fluxes up to around 900 photons per pixel per frame (20 keV equivalent) as measured by DynamiX. Three different calibrations were tried: #2*A* is the calibration used in Fig. 20 ▸, where 1*F* (fine count) = 1 keV and 1*C* (coarse count) = 64*F*. Calibration #2*B* also targets 1*F* = 1 keV and 1*C* = 64*F*, but with a fresh calibration directly before these X-ray set measurements were taken, and with the coarse threshold set at exactly that corresponding to the mean of the S curve (Fig. 17 ▸), *not* a little higher, as in #2*A*. The coarse cancellation packet size was therefore also slightly different after the procedure in Fig. 18 ▸ was carried out again. Calibration #1 uses this alternative calibration procedure, but with 1*F* = 6 keV and 1*C* = 64*F*, which is closer to the typical use case of DynamiX.

Excellent linearity is seen for all three calibrations, with r.m.s. linearity < 3.5% for all three. Note that calibration #1 causes the coarse counter to saturate at the highest fluxes. The ratio of the gradients of #2*A* to #1 is approximately 6.1, and #2*B* to #1 is 5.5. The ideal ratio is 5.9 (the ratio of the fine count to kiloelectronvolts conversions corresponding to 100DAC and 700DAC in Fig. 14 ▸). There is a ∼11% difference in the gradients between calibrations #2*A* and #2*B* due to the slightly different coarse threshold and cancellation packet size, which resulted from both a new calibration and a slightly different calibration procedure. Without an independent measurement of flux, it is not possible to say which calibration is more accurate.

### 5.6. Imaging a spinning slitted disc with the whole array

For these measurements the ‘X-ray set (imaging)’ setup was used, as shown in Fig. 6 ▸. The data were flat-field corrected to account for both the offsets between pixel thresholds and inter-pixel variation between charge cancellation packet sizes described in Section 2. The flat-field-corrected image *I* was calculated (Van Nieuwenhove *et al.*, 2015 ▸) as
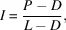
where *P* is the image with sample, *D* is the dark image without X-rays and *L* is the flat-field image with X-rays but no sample.

**Figure 23:**
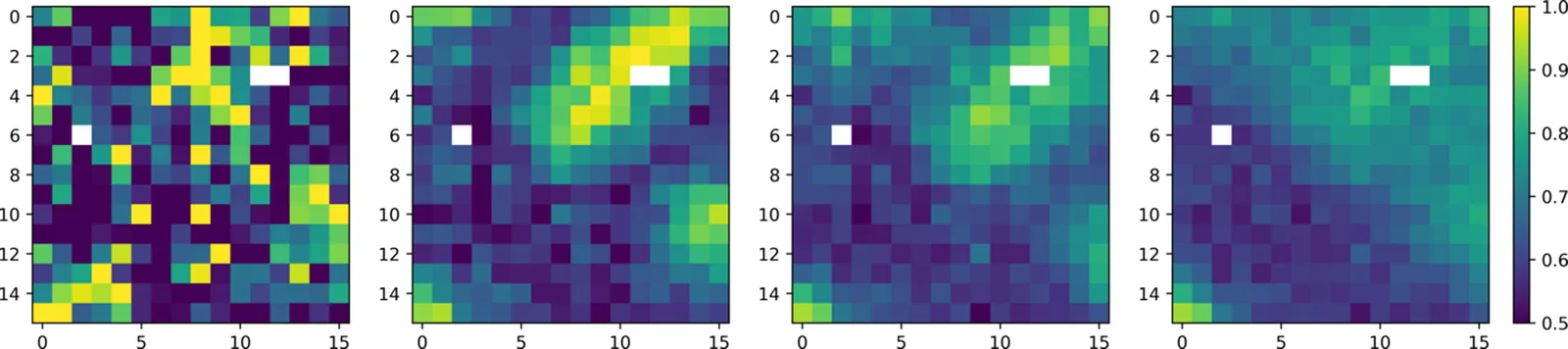
Full array X-ray images of a spinning slitted disc, after dark- and flat- field correction and averaging over (left to right) 1, 20, 50 and 100 raw frames. The openings in the slits, the brighter regions in yellow in the central left figure, are decreasingly visible at longer averaging due to movement blur.

The detector was moved far away from the X-ray set for this imaging measurement, which is intended as a qualitative demonstration of the array’s performance. Relative to the measurements shown in Fig. 21 ▸, the flux was therefore significantly reduced. Furthermore, the tube voltage was reduced from 160 kV to 30 kV to increase attenuation in the thin copper and steel and therefore contrast in the image, but this simultaneously reduced the flux. The flat-field-corrected images (using fine counts only), averaged over 1, 20, 50 and 100 frames, are shown in Fig. 23 ▸, and an animation is included in the supporting information, demonstrating imaging with DynamiX at a 0.5 MHz frame rate. Pixels that do not work correctly, either due to a faulty ASIC channel, a poor bond from the ASIC pad to the CZT pixel, or imperfections in the CZT bulk or contact, are shown in white. The flux and ASIC parameters were not optimized for this measurement, so the noise in the single-frame average, and the dark regions, may be partially attributed to shot noise from the relatively low flux. Further measurements are planned to optimize the setup.

## 6. Conclusions and next steps

Results from the DynamiX test structure have been presented, showing a noise performance in typical use of around σ = 5.7 ± 0.1 keV (with a fine-charge packet size of ∼1 keV) and r.m.s. linearity < 7% over more than three orders of magnitude of flux, from <1 photon per pixel per frame up to >900 photons per pixel per frame equivalent at 20 keV.

The firmware and software can robustly capture packets, assemble full 16×16 pixel frames and save to disk at 534000 frames per second. These measurements have also confirmed the presence of an ‘excess leakage current’ effect in HF-CdZnTe at high fluxes which has been seen previously by others.

Testing has revealed areas for improvement in the chip design, including introducing trims to minimize per-pixel variation. To design the full-scale ASIC, the DynamiX team has formed a collaboration with the XIDER team at Heidelberg University and ESRF, to develop XIDyn, a 192×144 pixel detector.

## Supplementary Material

GIF animation of Fig. 23. DOI: 10.1107/S1600577526006375/tol5025sup1.gif

## Data Availability

Data may be accessed upon request.
